# UV Irradiation Induces a Non-coding RNA that Functionally Opposes the Protein Encoded by the Same Gene

**DOI:** 10.1016/j.cell.2017.01.019

**Published:** 2017-02-23

**Authors:** Laura Williamson, Marco Saponaro, Stefan Boeing, Philip East, Richard Mitter, Theodoros Kantidakis, Gavin P. Kelly, Anna Lobley, Jane Walker, Bradley Spencer-Dene, Michael Howell, Aengus Stewart, Jesper Q. Svejstrup

**Affiliations:** 1 Mechanisms of Transcription Laboratory, The Francis Crick Institute, Clare Hall Laboratories, South Mimms EN6 3LD, UK; 2 Institute of Cancer and Genomic Sciences, University of Birmingham, Vincent Drive, Edgbaston, Birmingham B15 2TT, UK; 3 Bioinformatics and Biostatistics, The Francis Crick Institute, 1 Midland Road, London NW1 1AT, UK; 4 Experimental Histopathology, The Francis Crick Institute, 1 Midland Road, London NW1 1AT, UK; 5 High Throughput Screening Laboratory, The Francis Crick Institute, 1 Midland Road, London NW1 1AT, UK

**Keywords:** DNA damage response, transcript elongation, ASCC3, non-coding RNA, lncRNA, alternative last exon splicing, UV-irradiation, RNA polymerase II

## Abstract

The transcription-related DNA damage response was analyzed on a genome-wide scale with great spatial and temporal resolution. Upon UV irradiation, a slowdown of transcript elongation and restriction of gene activity to the promoter-proximal ∼25 kb is observed. This is associated with a shift from expression of long mRNAs to shorter isoforms, incorporating alternative last exons (ALEs) that are more proximal to the transcription start site. Notably, this includes a shift from a protein-coding *ASCC3* mRNA to a shorter ALE isoform of which the RNA, rather than an encoded protein, is critical for the eventual recovery of transcription. The non-coding ASCC3 isoform counteracts the function of the protein-coding isoform, indicating crosstalk between them. Thus, the *ASCC3* gene expresses both coding and non-coding transcript isoforms with opposite effects on transcription recovery after UV-induced DNA damage.

## Highlights

• UV elicits elongation slowdown and restricts transcription to the 5′ end of genes
• UV induces a switch from long to short alternative last exon (ALE) transcript isoforms
• *ASCC3* short and long ALE isoforms have antagonistic functions in the UV response
• The UV-induced ASCC3 short isoform functions as a long non-coding RNA

## Graphical Abstract

**Figure:**
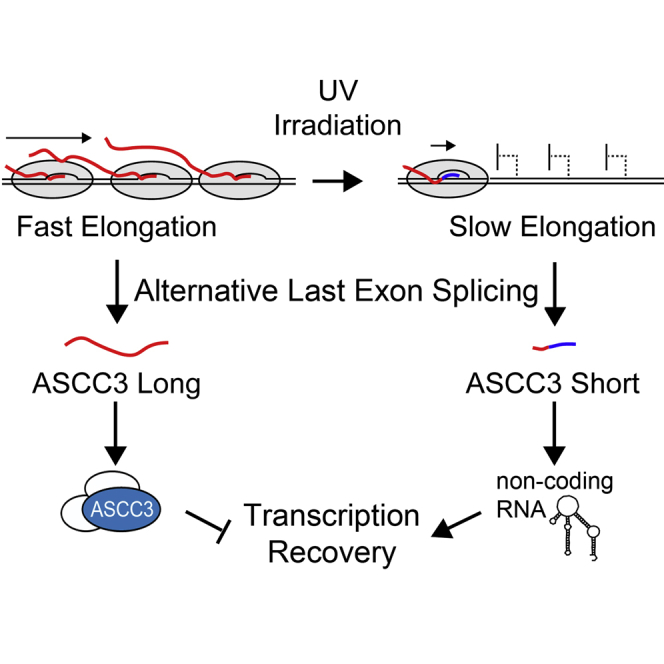

## Teaser

UV damage generates a functional non-coding RNA through alternative pre-mRNA processing of a damage response factor transcript, identifying a pathway for repurposing protein coding genes under selective conditions.

## Introduction

The efficient production and correct processing of nascent RNA polymerase II transcripts is essential for life. Factors that affect transcription and mRNA splicing, including DNA damaging agents, can thus have a dramatic effect on gene expression and cell viability. Indeed, bulky DNA lesions such as those generated by UV irradiation trigger rapid shutdown of RNA synthesis (Mayne and Lehmann, 1982, Rockx et al., 2000). They also elicit transcription-coupled repair (Gaillard and Aguilera, 2013), and, as a last resort, ubiquitylation and degradation of damage-stalled RNA polymerase II (RNAPII) (Wilson et al., 2013).

Although both transcriptional initiation and elongation are affected by UV irradiation (Rockx et al., 2000, Proietti-De-Santis et al., 2006, Andrade-Lima et al., 2015), the extent, mechanism and functional consequence of the changes occurring in these processes remain poorly understood. UV irradiation induces global changes to RNAPII phosphorylation (Rockx et al., 2000), altered binding of TATA-binding protein to DNA (Vichi et al., 1997), and modifications to chromatin (Adam et al., 2013, Dinant et al., 2013), underscoring the complexity of the transcription-related DNA damage response. Moreover, transcription-repair coupling factor Cockayne syndrome B (CSB) is required not only for DNA repair, but also for transcription restart after DNA damage (Proietti-De-Santis et al., 2006).

The vast majority of RNAPII genes have the potential to be expressed as multiple mRNA isoforms, creating vast regulatory potential (Pan et al., 2008, Wang et al., 2008). Indeed, changes in alternative isoform expression can regulate the physiological response of cells to stress or other signals. Importantly, processing of nascent pre-mRNA occurs co-transcriptionally, so that mRNA capping, splicing, and 3′ end formation are greatly influenced by the dynamics of elongation (de la Mata et al., 2003, Ip et al., 2011, Pinto et al., 2011, Fong et al., 2014). A general kinetic model has hence emerged wherein the rate of elongation governs RNA processing (de la Mata et al., 2003, Muñoz et al., 2009, Pinto et al., 2011).

To better understand the effect of UV irradiation on gene expression, we examined nascent transcription and transcript isoform expression on a genome-wide level. We hereby uncovered evidence that UV-induced alternative last exon (ALE) splicing is important for the DNA damage response, with long and short *ASCC3* ALE isoforms having opposite effects on transcription recovery after DNA damage. We also show that the short ASCC3 isoform regulates transcription recovery in a manner that is dependent on the non-coding RNA rather than the encoded protein.

## Results

### Transcript Elongation Rates Are Reduced Immediately after UV Irradiation

**Figure 1:**
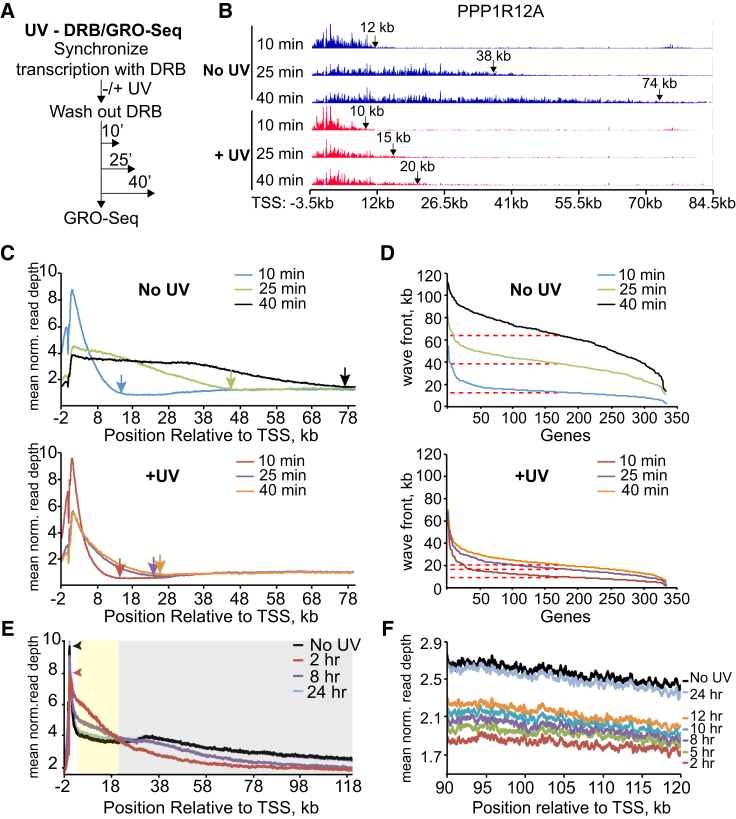
UV Irradiation Triggers Transcript Elongation Slow-Down (A) Schematic of UV/DRB/GRO-seq. (B) Profile of nascent PPP1R12A RNA reads after DRB-release ± UV irradiation (15 J/m^2^ used throughout this study). Arrows indicate transcription wave-fronts. (C) As in (B), but meta-gene profile of normalized GRO-seq reads across 8,148 genes. (D) Position of the GRO-seq transcription wave-front for 333 long genes over time ± UV irradiation. Dashed lines indicate median wave-front positions. (E) Meta-gene profile of normalized GRO-seq reads −2 kb to +120 kb relative to the TSS, ± UV irradiation followed by 2, 8, and 24 hr recovery. Arrows indicate the height of the promoter proximal peak. Shaded areas indicate gene regions characterized by increased (yellow) or decreased (gray) GRO-seq signal 2 hr after UV exposure, normalizing over time. (F) Gradual recovery of GRO-seq reads 90–120 kb downstream of the TSS following UV irradiation. See also Figure S1.

To investigate the effect of UV irradiation on transcription genome-wide, we employed 5,6-dichloro-1-β-D-ribofuranosylbenzimidazole/global run-on sequencing (DRB/GRO-seq), which allows measurement of nascent RNA synthesis at a high temporal and spatial resolution (Saponaro et al., 2014). Cells were first treated with the transcription elongation inhibitor DRB to restrict RNAPII to the promoter-proximal areas (first ∼600 bp of genes). Cells were then UV-irradiated, followed by inhibitor removal to allow synchronized transcription and its genome-wide measurement by GRO-seq (Figure 1A). Results from the PPP1R12A gene are shown as an example (Figure 1B). In untreated cells, RNAPII progressed ∼12 kb into the gene 10 min after DRB removal and to ∼38 kb and ∼74 kb after 25 and 40 min, respectively. These results mirror previously published data (Saponaro et al., 2014), but were in striking contrast to those obtained when cells were UV-irradiated before DRB removal. Here, the position of the RNAPII “wave-front” was similar to that of untreated cells after 10 min. However, a dramatic reduction in RNAPII progress was observed 25 and 40 min after UV exposure, with the wave-fronts in the PPP1R12A gene moving only very slightly further forward, reaching ∼15 and ∼20 kb at these time points (Figure 1B). We note that little change was observed at the promoter at these times. DRB/GRO-seq only captures the activity of RNAPII molecules that incorporate 5-bromouridine-5′-triphosphate (Br-UTP) during the short run-on pulse (5 min). This suggests that initiation and transcript elongation in the promoter-proximal areas still occurred, while progress further into genes was very slow or prohibited.

Meta-gene profiles of 8,148 transcripts revealed that UV irradiation generally attenuated elongation markedly, with nascent RNA wave-fronts reaching ∼75 kb after 40 min in untreated cells (Figure 1C, upper, black arrow), but only ∼25 kb after UV irradiation (Figure 1C, lower, orange arrow).

**Figure S1:**
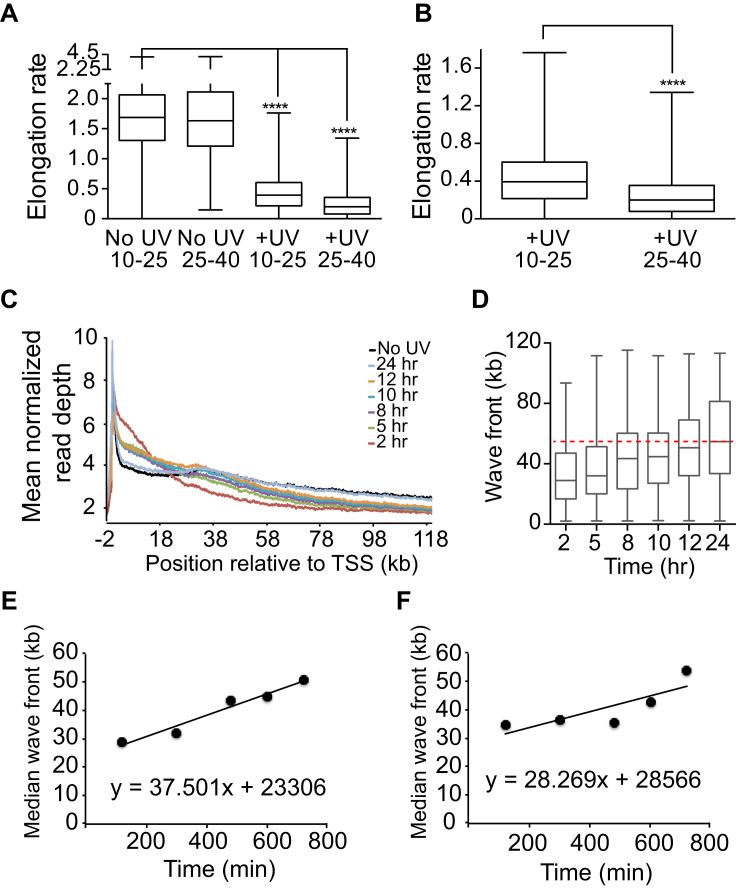
Transcription Wave-Front and Elongation Rates, Related to Figure 1 (A) Elongation rate (kb/min) calculated for 333 long genes for the indicated time intervals in untreated and UV-treated cells. Data are box and whisker (min to max) with median indicated. Mann-Whitney test, ^∗∗∗∗^p < 0.0001. (B) Comparison of the elongation rate for the 10-25 and 25-40 min time intervals in UV-treated cells. (C) Meta gene profile across the region −2 kb to 120 kb relative to the TSS of mean normalized read GRO Seq read density from untreated cells (No UV) and cells treated with UV followed by 2, 5, 8, 10, 12 and 24 hr. (D) Computationally determined wave fronts for 141 genes during transcription recovery, 2-24 hr post-UV. Data are box and whisker (min to max), with median indicated. The median wave front for the 24 hr time point is indicated by the red dashed line. (E). The median wave fronts from B were plotted for samples 2-12 hr after UV treatment. A line of best fit and the corresponding equation is shown. The slope of the line indicates the rate of transcription elongation as determined by individual gene wave front calling. (F) Mathematically determined GRO-Seq transcription wave fronts during the transcription recovery 2-12 hr post-UV. A line of best fit and the corresponding equation is shown. The slope indicates the rate of transcription elongation as determined by mathematical wave front calling.

To calculate the UV-induced reduction in elongation rates, the nascent RNA wave-front was called for a subset of very long transcripts (n = 333) (Figure 1D). In untreated conditions, the wave-front progressed to a median distance of 12.5 kb after 10 min and to 39 kb and 64.8 kb after 25 min and 40 min, respectively (Figure 1D, upper; indicated by dashed lines). This corresponds to average elongation rates of 1.77 kb/min (10–25 min) and 1.72 kb/min (25–40 min). In contrast, in UV-treated cells (Figure 1D, lower), the wave-fronts were at 10.3 kb (10 min), 17.3 kb (25 min), and 21.0 kb (40 min), respectively (Figure 1D, lower), giving rise to average elongation rates of only 0.47 kb/min (10–25 min) and 0.25 kb/min (25–40 min) (see also Figures S1A and S1B).

### RNAPII Progresses Slowly during Transcription Restart after UV Irradiation

Based on experiments that measured nascent RNA synthesis by general radioactive labeling (Mayne and Lehmann, 1982, Rockx et al., 2000, Proietti-De-Santis et al., 2006), transcription levels should recover to near-normal levels over an ∼24-hr period. To analyze transcription restart genome-wide, we therefore performed GRO-seq experiments with cells that were again UV-irradiated at 15 J/m^2^, followed by recovery (Figures 1E and S1C). This dose of UV did not lead to significant cell death over the 24-hr time course (data not shown). As expected, the distribution of active RNAPII in untreated cells was characterized by a large peak in the promoter-proximal region, followed by a marked reduction in signal further downstream (black graph). Transcription was not synchronized with DRB, so this density pattern represents the distribution of RNAPII expected for actively transcribed genes at steady state. In response to UV irradiation (2 hr time point), there was a clear reduction in the promoter-proximal peak (see arrowheads in Figure 1E), suggesting either a reduction in transcription initiation or increased promoter clearance (Ehrensberger et al., 2013). Interestingly, the GRO-seq signal increased in the region up to ∼20 kb from the *transcription start site* (TSS) (Figure 1E, yellow shaded region), concomitant with depletion further downstream (Figures 1E, gray shaded region, and 1F). This suggests that while transcription initiation may be inhibited, considerable elongation activity is observed in the beginning of genes (possibly reflecting increased promoter release), and activity is dramatically reduced in regions further downstream.

As expected, RNA synthesis gradually normalized to that observed in untreated cells over the 24-hr period, with eventual restoration of activity at the 3′ end of genes (Figures 1E and 1F). Interestingly, wave-front calling of a subset of very long genes indicated a rate of transcript elongation of only ∼40 bases/min on average from 2 to 12 hr following UV irradiation, more than 40-fold slower than in untreated cells (Figures S1D and S1E). Mathematically determined, median transcription “wave-fronts” independently confirmed these results (Figure S1F).

Taken together, these data suggest that UV irradiation causes a rapid and dramatic reduction in transcript elongation, and even upon recovery of nascent RNA synthesis several hours after UV exposure, elongation continues to be much slower than in untreated cells. Most importantly, transcription is spatially restricted for long periods, with the promoter-proximal 20–25 kb showing much more activity than the areas further downstream.

### UV-Induced Alternative Isoform Expression

**Figure 2:**
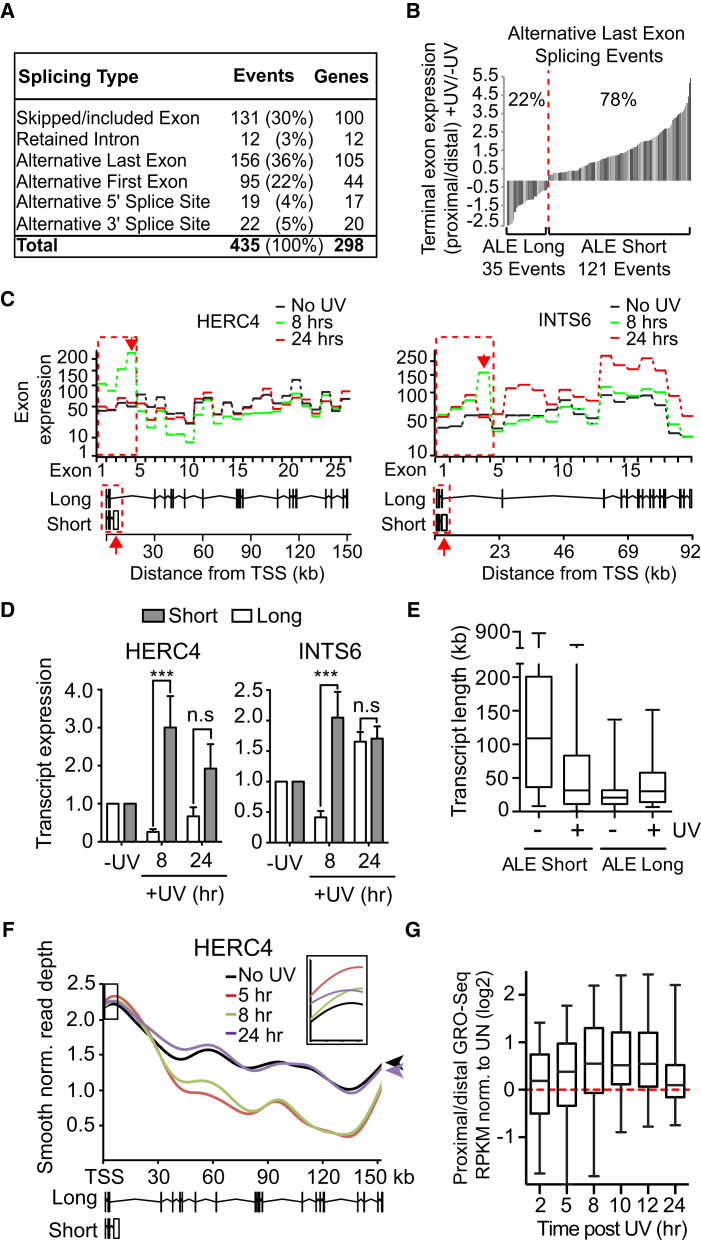
Splicing Analysis Reveals Frequent UV-Induced Alternative Last Exon Splicing (A) UV-induced splicing events. (B) Relative expression of terminal exons associated with UV-induced ALE splicing events. The ratio of proximal to distal terminal exon was calculated for the UV-treated sample and normalized to the control. (C) Exon expression profiles for *HERC4* and *INTS6* upon UV irradiation. Red, dashed box indicates exons associated with expression of the short isoform. Schematic illustrations are shown below. Red arrows indicate terminal exons specific to the short isoforms. (D) qRT-PCR validation of isoform expression. GAPDH normalized data relative to untreated conditions. (E) Change in pre-mRNA length of ALE short events (left) and long events (right). Box and whisker plots with min/max/median represent pre-mRNA lengths. (F) GRO-seq signal across *HERC4* after UV exposure (boxed inset, area of short isoform). Arrowheads highlight recovery of gene synthesis at the 3′ end after 24 hr. (G) Box and whisker plots (5–95 percentile with min/max/median indicated), showing relative GRO-seq read density of terminal exons following UV irradiation, normalized to untreated. Data for (D) and others like it in the following figures are mean ± SEM, t test, ^∗^p < 0.05, ^∗∗^p < 0.01, ^∗∗∗^p < 0.001 and ^∗∗∗∗^p < 0.0001. See also Figures S2 and S3 and Tables S1, S2, and S3.

Considering the dramatic change in transcript elongation and knowing that mRNA processing is tightly coupled to elongation, we now investigated the effect of UV irradiation on mRNA splicing by next generation sequencing of cDNA libraries generated from mRNA. The relative expression of transcript isoforms was quantitatively measured using the mixture of isoform (MISO) model (Katz et al., 2010). In total, we identified 435 splicing events in 298 genes that were affected either 8 or 24 hr after UV irradiation in both biological replicates (Figure 2A; Table S1).

Previous reports uncovered examples of increased inclusion of cassette exons under conditions of attenuated elongation (de la Mata et al., 2003, Muñoz et al., 2009, Fong et al., 2014). Our analysis of 131 UV-induced exon skipping/inclusion events shows only a slight bias (63% of events) for increased exon inclusion after UV irradiation (data not shown).

**Figure S2:**
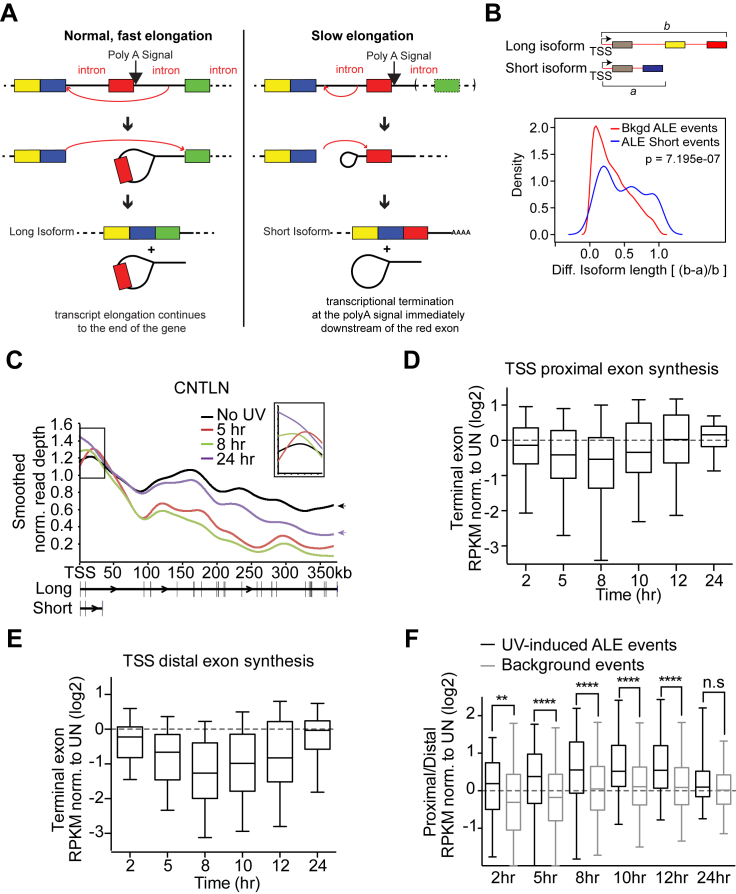
UV-Induced ALE Short Events Reduce Transcript Length and Correlate with Nascent RNA Synthesis Compared to Background Events, Related to Figure 2 (A) Diagram showing UV-induced alternative last exon splicing and premature termination. Normal, fast elongation favors the skipping of the red exon along with its associated poly-A signal, leading to preferential production of the long isoform. Slower elongation favors splicing of the red exon leading to utilization of the associated poly-A signal and expression of the short isoform. (B) Illustration depicts the variables used for the equation for relative change in isoform length ((b-a)/b). Plot of relative change in isoform length for ALE short events (blue line) (n = 121) compared to non-UV regulated background events (n = 7736) (red line) shows UV-regulated events are characterized by a statistically significant greater difference in short and long isoform length compared to events that were not effected by UV. Wilcox test, p = 7.195 X10^−7^. (C) Smoothed GRO-Seq signal across the CNTLN gene showing increased synthesis spanning the 5′ region gene corresponding to the short isoform (see boxed inset, 8 and 24 our after UV) and reduced signal across the rest of the gene corresponding to the long isoform. Arrows highlight the sustained repression of synthesis at the 3′ end of the gene 24 hr after UV correlating with preferential short isoform expression at this time point. (D and E) GRO-Seq read density mapped to the terminal exon of UV-induced short isoforms (D) and UV-suppressed long isoforms (E) at the indicated time points after UV-irradiation normalized to untreated samples. Data are box and whisker (min to max), with median indicated. (F) No increase of short over long isoforms was observed for ‘background’ ALE events that were unaffected by UV treatment. The ratio of short isoform (TSS proximal) to long isoform (TSS distal) terminal exon expression at the indicated times following UV treatment normalized to untreated is shown for background events not regulated by UV (n = 7736) (non-hits, gray boxes) and for UV-induced ALE short events (n = 121) (hits, black boxes). Data are box and whisker (min to max) with median indicated. Mann Whitney test, ^∗∗^p < 0.01, ^∗∗∗∗^p < 0.0001.

Interestingly, alternative last exon (ALE) splicing was the most frequent UV-induced event (Figure 2A; Table S2), accounting for more than a third of all those recorded: 156 ALE splicing events in 105 genes. ALE transcript isoforms are characterized by different 3′ terminal exons and therefore inherently have different poly-A sites. Importantly, a marked bias for expression of shorter transcript isoforms (induced ALE transcript isoforms that have terminal exons more proximal to the TSS) was observed following UV irradiation (Figure 2B), with 78% of ALE events (121 of 156) resulting in increased expression of such shorter isoforms (hereafter referred to as “ALE short” events). The majority of these (71/121) involved alternative splicing of unique terminal exons, indicating they were not solely a result of premature termination (Figure S2A). Only 35 events were characterized by increased expression of alternative longer isoforms, from 22 genes (Figure 2B, “ALE long” events).

The relative exon expression of the isoforms for two genes, *HERC4* and *INTS6*, is described in Figure 2C. The long *HERC4* pre-mRNA isoform contains 25 exons and is 153 kb, while the short *HERC4* pre-mRNA is 6.3 kb, shares the first three exons with the long isoform, but contains a fourth, unique terminal exon (Figure 2C, left, lower). Eight hours after UV irradiation, exons 1–4 (indicated by the red dashed boxes) were induced (Figure 2C, left). In contrast, expression of exons 5–26 (specific for the long isoform) was reduced, but recovered to near-normal levels after 24 hr. A similar pattern of alternative exon expression was seen at the *INTS6* gene and a large number of other genes (Figure 2C, right, and data not shown). qRT-PCR confirmed the increased expression of the short isoforms and concomitant lower expression of the long isoforms 8 hr after UV irradiation (Figure 2D).

The short RNA isoforms of *HERC4* and *INTS6* were much shorter than their long isoforms. More generally, the median length of the UV-suppressed long pre-mRNA isoforms was ∼109 kb, considerably longer than that of all human genes (23 kb), whereas that of the UV-induced short isoforms was only ∼32 kb. This reduction in pre-mRNA length for UV-induced ALE short events was significantly greater than expected by chance (Figure S2B), indicating a general trend for switching from particularly long isoforms to much shorter isoforms upon UV exposure. In contrast, the median length of the less common UV-induced long ALE isoforms was ∼30 kb, only 9 kb longer than the median length of their corresponding, UV-suppressed short isoform (Figure 2E).

### ALE Events Are Associated with Changes in RNAPII Elongation and Nascent RNA Synthesis

Because transcript elongation was attenuated after UV irradiation (Figure 1), we hypothesized that the UV-induced ALE short events resulted from preferential synthesis of the pre-mRNA producing them. Indeed, after UV-irradiation, GRO-seq read depth at *HERC4* increased over the region coding for the short isoform (Figure 2F, inset), whereas synthesis in the rest of the gene was markedly suppressed. Nascent RNA synthesis across the entire gene recovered to untreated levels 24 hr after UV exposure, correlating with the kinetics of the *HERC4* ALE splicing event. Consistent with a causative effect, the GRO-seq signal corresponding to the long isoform also remained suppressed at 24 hr for a gene in which preferential expression of the short isoform was detected not only at 8 but also 24 hr after UV irradiation (Figure S2C).

By comparing GRO-seq signals across proximal and distal terminal exons, a general, transient increase in the ratio of short to long transcript isoform expression was observed, peaking 8–12 hr after UV (Figure 2G). This increase correlated with a greater reduction in the synthesis of distal than of proximal exons and was specific for UV-induced ALE events (Figures S2D–S2F).

Together, the results presented so far indicate that UV irradiation results in a dramatic change in transcription, with elongation slowing down and RNAPII-mediated RNA synthesis being “restricted” to the 5′ end of genes. This gene-spatial restriction of transcription is associated with, or indeed causes, the preferential expression of short transcript isoforms incorporating alternative last exons.

### ASCC3 Short Isoform Is Preferentially Synthesized in Response to UV

**Figure S3:**
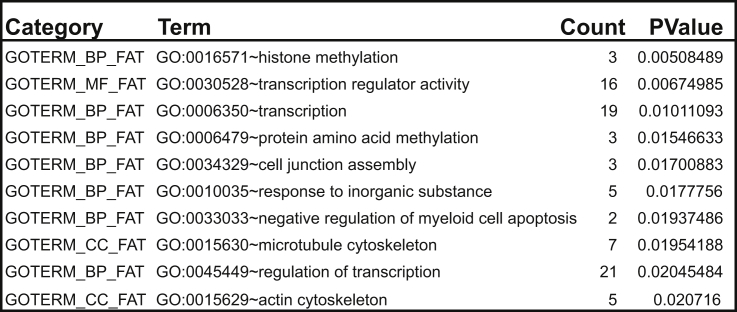
Gene Ontology Analysis of ALE Short Genes, Related to Figure 2 Gene ontology of ALE short genes reveals a significant enrichment in genes involved in transcription and transcription regulation.

We now investigated whether the preferential expression of short ALE isoforms in response to UV irradiation is physiologically important. Gene ontology analysis of the 84 genes that undergo UV-induced ALE short isoform switching revealed that many of them are involved in transcription (Figure S3). We also cross-referenced the genes with a recently compiled database of factors that function in the transcription-related DNA damage response (Boeing et al., 2016). Interestingly, genes with short ALE events were enriched among the highest scoring genes in this database (p = 0.0077; Kolmogorov-Smirnoff test), with 28 of the 84 genes being among the 15% highest scorers (Table S3). Among these factors, *ASCC3* stood out: it had *the* highest score in the multi-omic screening approach (Boeing et al., 2016).

**Figure 3:**
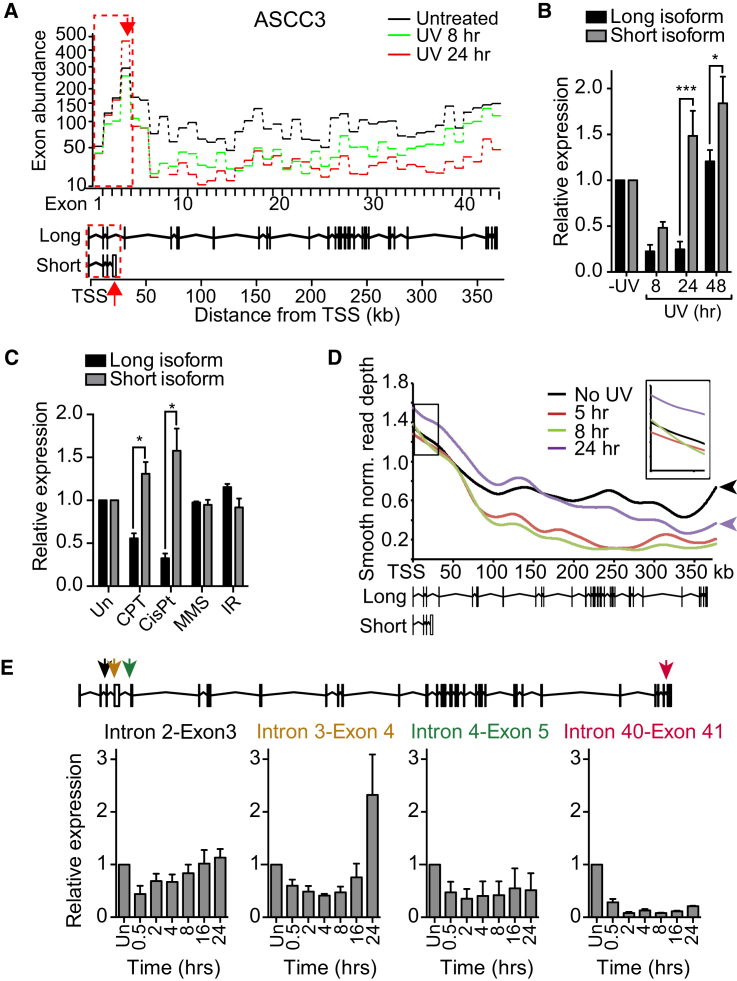
Bulky DNA Lesions Induce ASCC3 Alternative Last Exon Switching (A) ASCC3 exon expression profiles, as in Figure 2C. (B) qRT-PCR validation of the isoform switch 24 hr after UV irradiation. GAPDH normalized data relative to untreated conditions are averaged, ±SEM. (C) Expression, determined by qRT-PCR, of the different isoforms of ASCC3 upon exposure to 100 nM camptothecin (CPT), 20 μM cisplatin (CisPt), 0.001% MMS, or 5 Gy ionizing radiation (IR). Untreated conditions (Un) set to 1. GAPDH normalized as in (B). (D) GRO-seq signal across *ASCC3*, as in Figure 2F. (E) qRT-PCR of nascent pre-mRNA across *ASCC3* after UV irradiation, using intron-exon junction primers (averaged, 18S normalized data, shown relative to untreated).

The pre-mRNA giving rise to the long ASCC3 isoform is 373.5 kb and composed of 42 exons (Figure 3A). The short ASCC3 isoform is 25 kb in length and shares the first three exons with the long isoform, followed by a unique terminal exon (Figure 3A, last exon indicated by the red arrows). Exon expression for both isoforms was reduced 8 hr after UV treatment. However, 24 hr after UV treatment, expression of the exons of the short isoform increased while those specific for the long isoform remained repressed (Figure 3A). This result was confirmed by qRT-PCR (Figure 3B). Expression of the long isoform recovered by 48 hr after UV treatment. Exposure of cells to cisplatin and camptothecin, but not MMS or ionizing radiation, also resulted in preferential expression of the short ASCC3 isoform, indicating that this is a general response to agents inducing bulky DNA lesions (Figure 3C).

Similar to what was observed for *HERC4*, *INTS6*, and other genes, the increase in the short ASCC3 isoform was likely caused by the restriction of nascent RNA synthesis to the beginning of genes after UV irradiation (Figure 3D). Indeed, recovery of nascent RNA synthesis was only observed over the first half of *ASCC3* 24 hr after UV treatment. More importantly, however, RNA synthesis across the relevant first 25 kb of the *ASCC3* gene was induced at 24 hr (Figure 3D, inset; see also 3E). The transcription characteristics at *ASCC3* thus again correlated with ALE switching and preferential production of the short ASCC3 isoform after UV irradiation.

### ASCC3 Protein Affects Transcription after UV Irradiation

**Figure 4:**
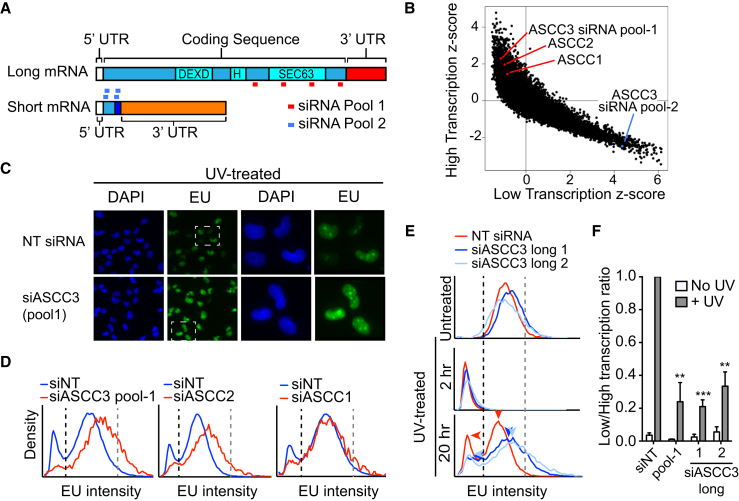
ASCC3 Long Isoform Knockdown Increases Global Transcription after UV Irradiation (A) ASCC3 isoforms and siRNA-targeting regions. Ninety-three nucleotide sequence not present in the long isoform shown in dark blue. (B) Scores from the genome-wide RNAi screen (Boeing et al., 2016) with ASCC3 (pool-1), ASCC2, and ASCC1 siRNA pools highlighted in red. ASCC3 (pool-2) is highlighted in blue. (C) Representative images of cells transfected with non-targeting (NT) siRNA and the ASCC3 siRNA pool-1 20 hr after UV irradiation. Nascent EU-labeled RNA shown in green and DAPI-stained nuclei in blue. 10× objective image on the left, with region in white box enlarged on the right. (D) Histogram plots of average EU incorporation following knockdown of ASCC1, 2 and 3 (pool-1) 20 hr after UV irradiation. Black and gray stippled lines demarcate thresholds of lowly and highly transcribing cells, respectively. (E) EU incorporation after treatment with NT siRNA (red) or individual siRNAs targeting ASCC3 long isoform (light and dark blue), with or without UV irradiation, measured after 2 and 20 hr. Data shown as in (D). Arrowheads highlight reduced proportion of lowly transcribing cells and shift of histogram to the right in cells lacking the long ASCC3 isoform. (F) The ratio of low to high transcribing cells (cells left of the black line over cells right of the gray line in E), in untreated conditions (white bars) or 20 hr after UV irradiation (gray bars). Data were averaged and normalized relative to UV-treated control cells (set to 1), ±SEM. See also Figure S4.

**Figure S4:**
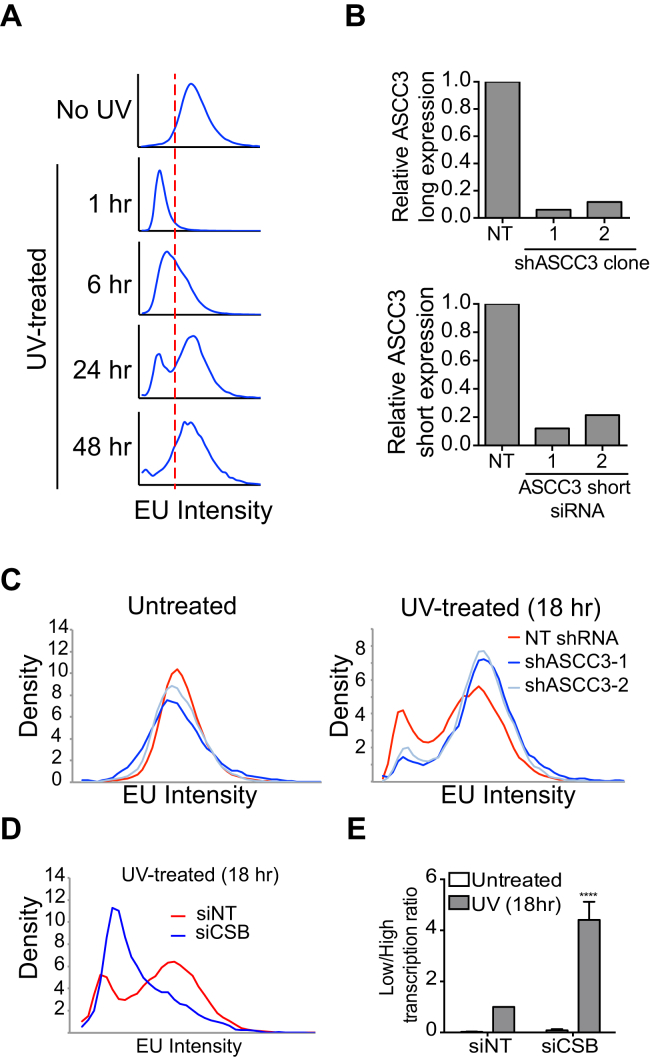
RNAi Knockdown of ASCC3 Isoforms and CSB and EU Labeling Assay, Related to Figure 4 (A) EU incorporation for untreated or UV-treated cell populations. Data are average EU intensity/nuclei displayed as histograms. There is a rapid reduction in EU incorporation 1 hr after UV treatment as indicated by a shift of the population to the left and corresponding increase in the percentage of cells below the low transcription threshold (indicated by the red dashed line). EU incorporation gradually recovers over the 48 hr time course. (B) RT-qPCR analysis of ASCC3 long isoform expression in cells stably expressing non-targeting (NT) shRNA and two clones stably expressing ASCC3-targeting shRNA (shASCC3) and short isoform expression 48 hr after transfection with NT and short isoform-targeting siRNA. (C) EU incorporation for shNT and shASCC3 cell lines in untreated (left panel) and UV-treated (15 J/m^2^, 18 hr recovery) (right panel) conditions. Data are average EU intensity/nuclei displayed as a histogram. (D) EU incorporation for UV-treated (15 J/m2, 18 hr recovery) NT and CSB-targeting siRNA transfected cells shows deficient recovery of transcription in CSB knockdown cells. Data are average EU intensity/nuclei displayed as a histogram. (E) The ratio of the proportion of low transcribing cells over high transcribing cells in untreated conditions (white bars) or 18 hr after UV (gray bars) transfected with NT or CSB siRNA. Data are mean -/+ SEM relative to UV-treated NT siRNA. t test, ^∗∗∗∗^p < 0.0001.

As expected from the results above, nascent RNA synthesis rapidly decreased after UV exposure as indicated by significantly reduced incorporation of ethynyluridine (EU) into nascent RNA, followed by a slow recovery (Figure S4A). In the multi-omic screening approach, we screened for nascent transcription using the EU incorporation assay to identify genes whose small interfering RNA (siRNA) knockdown affect transcription 20 hr after UV irradiation (Boeing et al., 2016). Intriguingly, two distinct siRNA pools targeting ASCC3 scored in this screen; one resulted in high transcription while the other resulted in low transcription levels after UV irradiation. Gratifyingly, the distinct siRNA pools targeted different ASCC3 ALE isoforms (Figures 4A and 4B).

ASCC3 siRNA pool-1 specifically targets the long mRNA isoform, which encodes the full-length ASCC3 protein. Knockdown with this pool resulted in high transcription levels after UV irradiation, as indicated by a reduced percentage of lowly transcribing cells and an overall increase in EU incorporation signified by a shift of the histogram to the right (Figures 4C and 4D, left histogram). ASCC3 is a component of the poorly studied activating signal co-integrator 1 complex (Jung et al., 2002). *ASCC3* was also identified in a screen for genes affecting infection of West Nile virus in interferon (IFN)-β-treated human cells, with silencing of *ASCC3* resulting in upregulation of certain interferon-stimulated genes (Li et al., 2013). However, a role for ASCC3 as a global suppressor of transcription is both unexpected and exciting. Tellingly, siRNAs targeting two other members of the ASCC complex, ASCC1 and ASCC2, also resulted in increased nascent transcription after UV irradiation (Figures 4B and 4D, center and right histogram), suggesting that the ASCC complex functions as an entity to keep transcription repressed after DNA damage. Moreover, two individual ASCC3 siRNAs, as well as stable shRNA expression targeting the long isoform increased transcription 20 hr after DNA damage, but did not affect transcription in untreated cells, or the immediate transcription shutdown observed 2 hr after UV irradiation (Figures 4E, S4B, top panel, and S4C). The differential effect at 2 and 20 hr is important, as it shows that transcription is suppressed in two distinct ways during UV-induced DNA damage, namely rapid ASCC3-independent transcriptional repression, followed by continued ASCC3-dependent suppression in the later stages of the DNA damage response. To measure the effect of ASCC3 knockdown at 20 hr quantitatively, we calculated the proportion of cells that fail to recover transcription relative to the proportion of cells that have high levels of transcription after UV irradiation (Figure 4E, lower panel, populations to the left of the stippled black line and right of the gray line, respectively). In response to UV exposure, knockdown of the long isoform of ASCC3 significantly reduced this low/high transcription ratio (Figure 4F). We conclude that the ASCC3 protein, in the context of the ASCC complex, suppresses transcription specifically in the late stages of the cellular response to UV irradiation.

### The Short ASCC3 RNA Isoform Is Required to Recover Transcription after UV Irradiation

**Figure 5:**
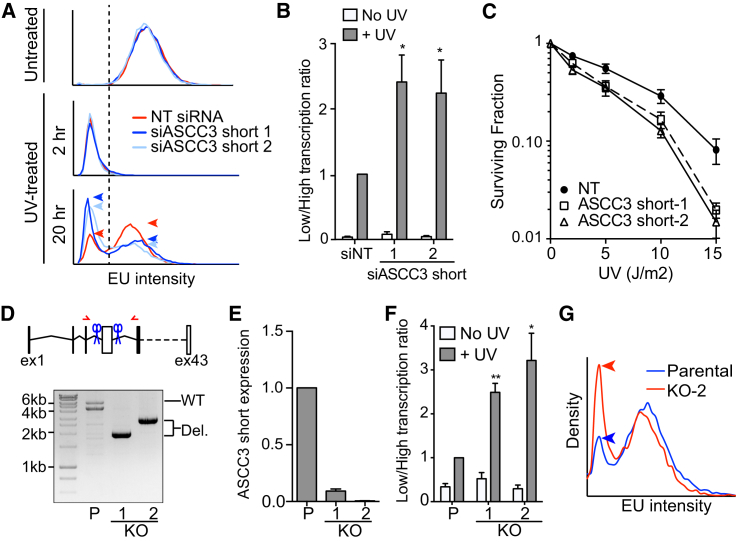
Cells Deficient for the Short ASCC3 Isoform Cannot Recover Transcription after UV Irradiation (A) As in Figure 4E, but after knockdown of the short ASCC3 isoform with individual siRNAs (light and dark blue). (B) As in Figure 4F, but after knockdown of short isoform. Data are mean ±SEM relative to UV-treated control. (C) UV-sensitivity measured by colony formation after knockdown with individual siRNAs targeting ASCC3 short isoform. Two-way ANOVA test: NT versus ASCC3 siRNA-1 p = 0.0182; NT versus ASCC3 siRNA-2 p = 0.008. (D) CRISPR-Cas9-mediated knockout of the unique, terminal exon of the short ASCC3 isoform. Genomic PCR fragments isolated from parental MRC5VA cells (P) and two knockout (KO) clones are shown (red arrows, primers; blue scissors, guide RNAs). (E) qRT-PCR analysis of short isoform RNA expression in the cell lines from (D), showing averaged GAPDH-normalized data, relative to parental cells. (F) Transcription recovery after deletion of the short ASCC3 isoform, measured as in Figures 4F and 5B. (G) Histograms showing decreased EU intensity/nucleus in ASCC3 short isoform KO clone-2 cells compared to control cells 20 hr after UV irradiation. See also Figure S5.

In marked contrast to siRNA pool-1, ASCC3 siRNA pool-2 dramatically reduced transcription after UV irradiation (Figure 4B). Two of the four siRNAs in pool-2 specifically target sequences unique to the terminal exon of the short alternative transcript isoform (Figure 4A, dark blue box), reducing short isoform transcript levels 79% and 82%, respectively (Figure S4B, lower panel). Knockdown with these siRNAs neither affected transcription in untreated cells, nor did it affect global transcription shutdown immediately after UV irradiation (Figure 5A, top and middle panels). However, in a manner similar to knockdown of Cockayne syndrome B (Figures S4D and S4E), knockdown of ASCC3 short isoform inhibited transcription recovery, as indicated by a marked general change in the characteristics of nascent transcription across the cell population (Figure 5A, 20 hr panel), and consequently an increase in the ratio of lowly to highly transcribing cells after UV irradiation (Figure 5B). Furthermore, knockdown of ASCC3 resulted in increased sensitivity to UV-irradiation (Figure 5C). This indicates that UV-induced expression of the short ASCC3 ALE isoform is indeed physiologically important, in all likelihood because this isoform is required for transcription to recover after UV irradiation.

**Figure S5:**
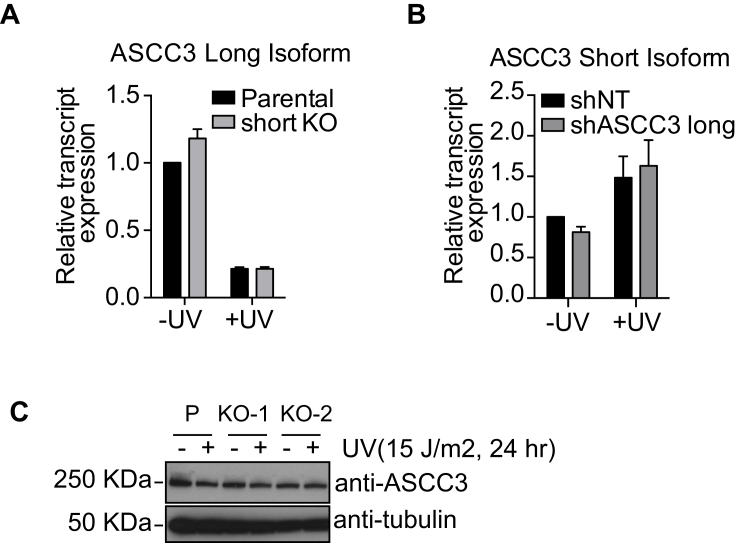
Deficiency in ASCC3 Short Isoform Does Not Affect ASCC3 Long Isoform Expression and Vice Versa, Related to Figure 5 (A) ASCC3 short isoform knockout cells that were left untreated or UV treated (15 J/m2) followed by 20 hr recovery were analyzed for long isoform expression by RT-qPCR. Data are normalized to GAPDH and relative to untreated parental samples, mean -/+ SEM. Knockout cells have a slight increase in long isoform expression (∼1.2 fold) in untreated conditions compared to parental controls but equally downregulate long isoform expression in response to UV. (B). Cells stably expressing ASCC3 long isoform-targeting shRNA or NT shRNA control were left untreated or UV treated (15 J/m2) followed by 20 hr recovery and analyzed for short isoform expression by RT-qPCR. Data are GAPDH normalized and relative to untreated shNT cells, mean -/+ SEM C. Expression of ASCC3 protein in ASCC3 short knockout cells and parental control cells in untreated and UV-treated (15 J/m^2^, 24 hr) conditions. Tubulin is shown as a loading control. Expression of ASCC3 protein is unaffected by short isoform knockout.

To confirm the role for the short ASCC3 isoform in transcription recovery, we also used CRISPR-Cas9-mediated gene editing to specifically remove the ALE that is specific to the short isoform, thereby abolishing short isoform expression but leaving the long isoform intact (Figures 5D, 5E, S5A, and S5C). As expected, these knockout cells, hereafter abbreviated “short knockout cells,” also showed a defect in transcription recovery in response to UV (Figures 5F and 5G).

### Antagonistic Regulation by the Short and Long *ASCC3* Isoforms

**Figure 6:**
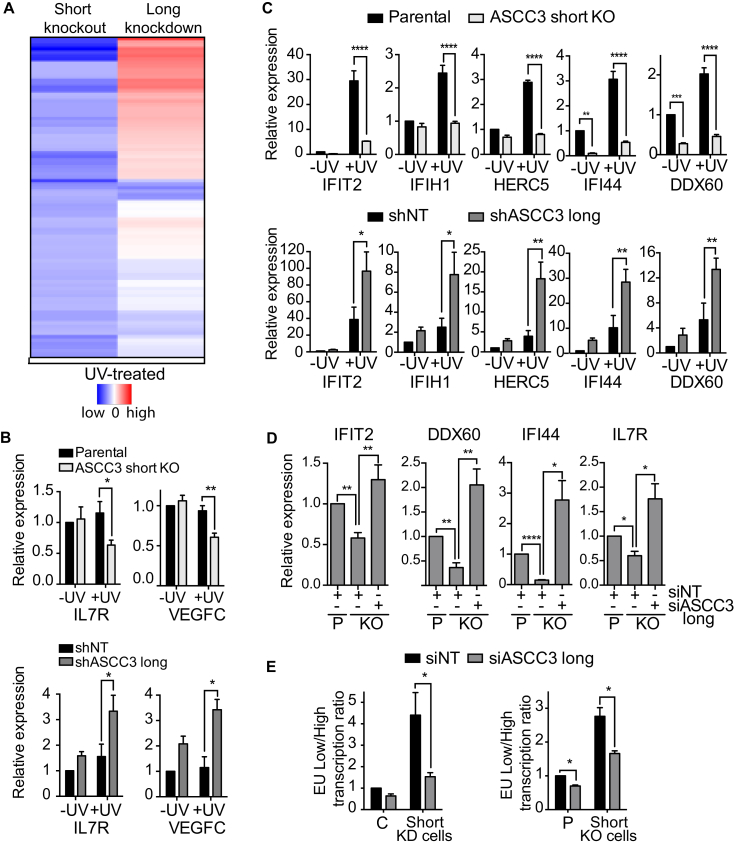
Numerous UV-Regulated Genes Are Antagonistically Regulated by the Long and Short ASCC3 Isoforms (A) Hierarchical clustering of genes downregulated in UV-treated ASCC3 short knockout cells, shown alongside expression of the same genes in UV-treated ASCC3 Long knockdown cells, relative to expression in control cells. Blue and red bars indicate minimum and maximum log fold-changes, respectively. (B) qRT-PCR analysis of *IL7R* and *VEGFC* expression in UV-treated short KO cells (light gray; top panel) compared to parental cells and in UV-treated long knockdown cells (dark gray; bottom panel) compared to NT shRNA cells (black) 20 hr after UV, shown as averaged GAPDH-normalized data, relative to untreated controls. (C) As in (B) but analysis of genes with UV-induced expression. (D) Rescue of gene expression in UV-treated short KO cells by transfection with siRNA targeting the long isoform. Analysis by qRT-PCR, with GAPDH-normalized data shown relative to UV-treated control cells. (E) As in (D) but for global nascent transcription, as indicated by the low/high transcription ratio 20 hr after UV irradiation, relative to control cells. C, siNT control; P, parental control. See also Figure S6 and Table S4.

In the analysis above, we focused entirely on nascent RNAPII transcription. To further characterize the role of the *ASCC3* isoforms in transcription after UV irradiation, we now used Illumina BeadArrays to compare their effect on stable mRNA expression 20 hr after UV irradiation. Compared to UV-treated control cells, 108 genes were differentially expressed in short knockout cells at this time-point, the majority of which (73%, 79/108) were downregulated (Figure 6A; Table S4). In contrast, 170 genes were differentially regulated in cells deficient for ASCC3 long isoform (long knockdown cell), of which 64% (107 genes) were upregulated. Interestingly, many of the genes that were downregulated in short knockout cells were upregulated in long knockdown cells (Figure 6A; p value < 10^−5,^ hypergeometric test on differentially regulated probes). qRT-PCR analysis of two such genes, *IL7R* and *VEGFC*, is shown in Figure 6B.

We also noticed that a subset of the genes that were most markedly affected by *ASCC3* were in fact greatly induced 20 hr after UV irradiation in control cells. Indeed, the increased expression of five such genes was largely eliminated in short knockout cells (Figure 6C, upper panels). Strikingly, all of these genes were “over-induced” in long knockdown cells (Figure 6C lower panels), again pointing to opposite regulatory effects of the long and short ASCC3 RNA isoforms.

**Figure S6:**
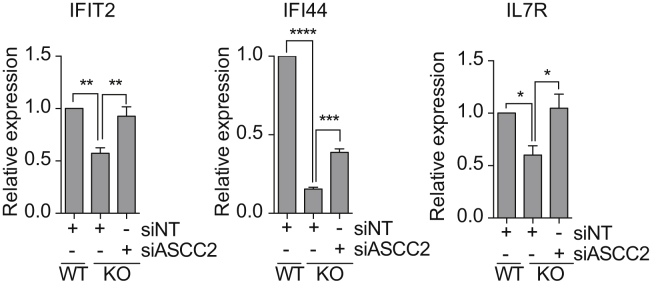
Rescue of Transcription in UV-Treated ASCC3 Short Isoform Knockout Cells by Knockdown of ASCC2, Related to Figure 6 ASCC3 short isoform knockout cells (clone 2) and parental cells (WT) were transfected with NT or ASCC2-targeting siRNA and incubated for 48 hr prior to exposure with UV (15 J/m^2^). Expression of IFIT2, IFI44 and IL7R was analyzed by RT-qPCR 20 hr after UV treatment. Data are GAPDH normalized and relative to UV-treated NT siRNA transfected parental cells, mean -/+ SEM. Note that the NT siRNA transfected samples are the same as those shown in Figure 6E. t test, ^∗^p < 0.05, ^∗∗^p < 0.01, ^∗∗∗^p < 0.001, ^∗∗∗∗^p < 0.0001.

The results presented so far suggest that the long and short ASCC3 isoform are functionally antagonistic: the ASCC complex (of which ASCC3 is a component) maintains transcriptional repression after DNA damage, while the short ASCC3 isoform seems to de-repress it. This raised the intriguing possibility that transcription defect observed in *ASCC3* short knockout cells might be rescued by depleting the long isoform. Strikingly, knockdown of the long ASCC3 isoform (Figure 6D), or ASCC2 (Figure S6), did indeed rescue the expression of several genes in short knockout cells following UV irradiation. Moreover, it also rescued the defect in global, nascent transcription recovery after UV irradiation, with the high proportion of lowly transcribing cells observed upon short ASCC3 knockdown (KD) or knockout (KO) returning to more normal levels when the long isoform was also depleted (Figure 6E). Importantly, knockdown of the long isoform did not affect expression of the short RNA isoform and vice versa (Figures S5A and S5B), showing that simple regulation of each other’s expression cannot underlie the antagonistic effects observed.

Together, these results support the idea that the long and short ASCC3 isoforms have opposing regulatory roles in transcription, affecting both global nascent transcription and stable mRNA expression of several individual genes in opposite directions.

### ASCC3 Short Isoform Functions as a Non-coding RNA

**Figure 7:**
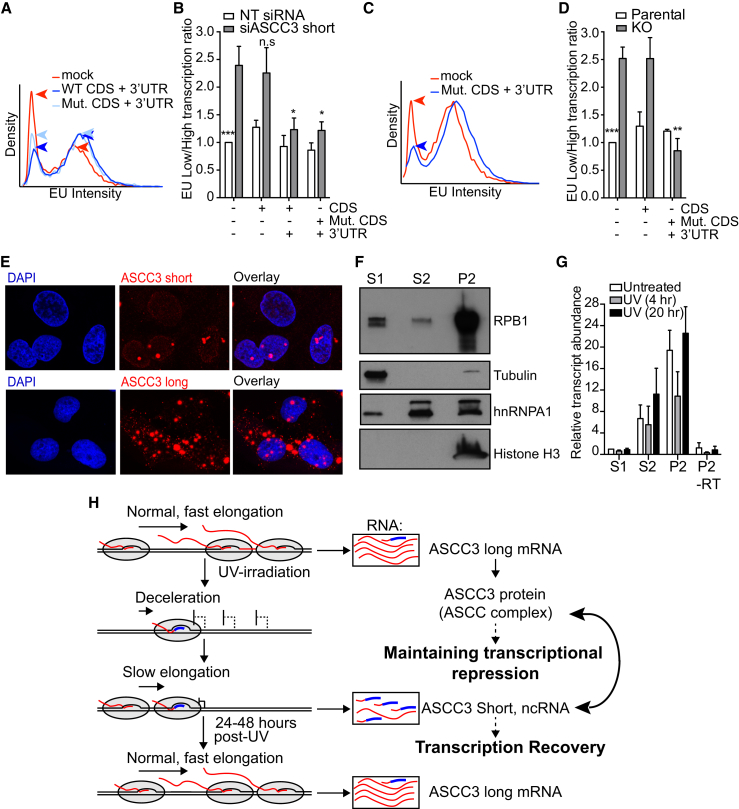
The Short ASCC3 Isoform Is a Chromatin-Associated lncRNA (A and B) Histogram (A) and low/high transcription ratio plot (B), showing the effect on transcription of expressing different siRNA-resistant RNAs in ASCC3 short isoform knockdown cells. (A) Blue arrows indicate the reduction in lowly transcribing cells and concomitant increase in highly transcribing cells following rescue with ASCC3 short isoform constructs containing the 3′UTR. Data in (B) are relative to UV-treated control cells, mean ± SEM. n.s., not significant; CDS, coding sequence; Mut. CDS, stop-containing CDS mutant. (C and D) As in (A) and (B) but for short isoform knockout cells. (E) RNA scope In situ hybridization signals for endogenous ASCC3 long and short isoforms. RNA scope signal (red) was overlaid with DAPI to highlight nuclear localization. (F) Immunoblot showing localization of RNAPII (RPB1 subunit), hnRNPA1, tubulin, and histone H3 following sub-cellular fractionation. S1, cytoplasmic; S2, soluble nuclear material; P2, chromatin pellet. (G) Enrichment of the short ASCC3 isoform in the S2 and P2 fractions as determined by qRT-PCR. As control, P2 was analyzed without reverse transcriptase (−RT). Data are relative to untreated S1 fraction, mean ± SEM. (H) Model showing RNAPII (gray sphere) producing nascent ASCC3 transcript (red), including the alternative last exon (thick blue line). Splicing determines exclusion/inclusion of the ALE and 3′-UTR (boxes on right). The protein-encoding long isoform mRNA and the non-coding short isoform have opposite effects on the DNA damage response and affect each other’s function (indicated by double arrow on right). See also Figure S7.

**Figure S7:**
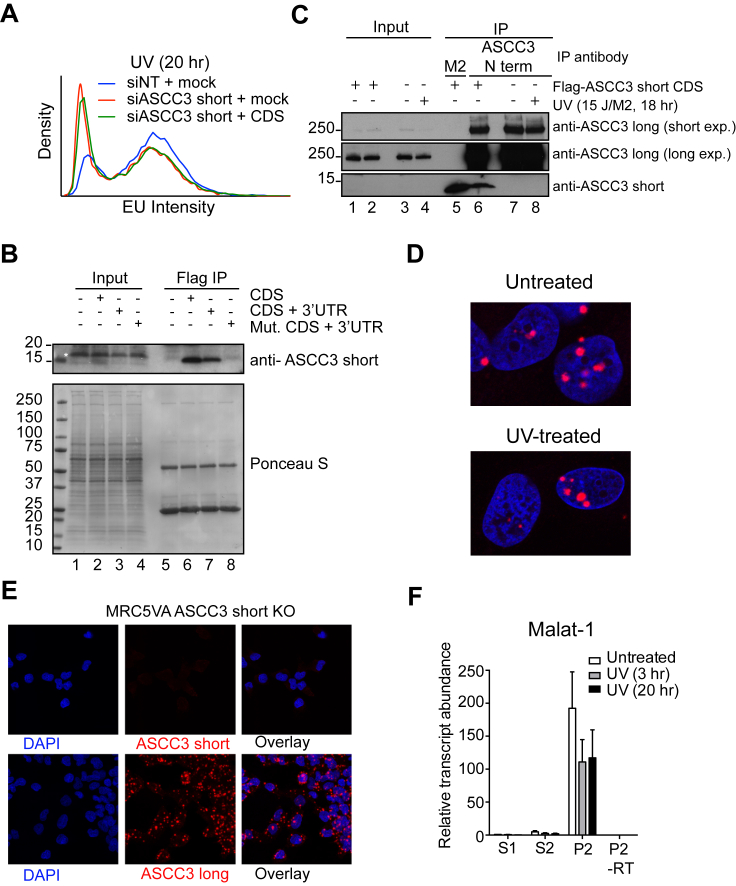
ASCC3 Short Isoform Transgene Expression and RNA Localization in MRC5VA Cells, Related to Figure 7 (A) Cells transfected with an ASCC3 short isoform coding sequence (CDS) expressing construct or mock transfected were incubated for 30 hr prior to transfection with NT or ASCC3 short isoform-targeting siRNA. Cells were UV-treated 48 hr after siRNA transfection followed by a 20 hr recovery. Data are average EU intensity/nuclei displayed in a histogram. Expression of ASCC3 short isoform CDS did not rescue the low transcription following ASCC3 short isoform knockdown. (B) Inputs (5%) (lanes 1-4) and M2 Flag immunoprecipitation (IP) (lanes 5-8) from untransfected cells and cells ectopically expressing Flag-tagged ASCC3 transcripts containing the coding sequence alone (CDS), coding sequence with 3′ UTR (CDS +3′UTR) and coding sequence with 3′UTR that contains an in-frame premature stop mutation (Mut. CDS +3′UTR). Immunoblot was probed with ASCC3 short isoform specific antibody. ASCC3 short isoform was not detected in input samples therefore Ponceau S stain is shown as a control for equal protein loading. ^∗^ indicates a non-specific band in input samples not present in IP elutions. (C) Inputs (10%) (lanes 1-4) and IP with anti-Flag M2 (lane 5) and ASCC3 N-terminal targeting antibodies (lanes 6-8) was performed on untransfected cells and cells transfected with constructs encoding Flag-tagged ASCC3 short isoform followed by immunoblotting using ASCC3 short and long isoform specific antibodies. Ectopically expressed short isoform was pulled down by both Flag and ASCC3 N-terminal antibodies (lanes 5 and 6) however short isoform protein was not detected in ASCC3 N-terminal antibody pull downs from untransfected cells under both untreated and UV-treated conditions (lanes 7 and 8). IP of ASCC3 long isoform is shown as a positive control for efficient N-terminal antibody pull down. ASCC3 short isoform was not detected in input samples. (D) RNA scope In situ hybridization followed by Fast Red staining using probes targeting the ASCC3 short isoform, in untreated cells and UV-treated cells after 18 hr recovery. The nucleus was counterstained with DAPI. The primarily punctate nuclear localization of ASCC3 was not significantly affected by UV treatment over several independent experiments. (E) RNA scope In situ hybridization in short isoform knockout cells with short and long isoform-targeting probes shows specific loss of short isoform signal but not long isoform signal confirming the short isoform probe signal was indeed specific for the short isoform of ASCC3. (F) LncRNA Malat-1 is enriched in the chromatin-associated P2 fractions as determined by RT-qPCR. The P2 fraction was also analyzed in the absence of reverse transcriptase (-RT) as a control. Data are relative to untreated S1 fraction, mean -/+ SEM.

The UV-induced short mRNA isoform contains a 333 nt coding sequence (CDS), the protein product of which is only 13 kDa and lacks known functional domains (see Figure 4A). Frustratingly, ectopic expression of this CDS failed to suppress the low transcription phenotype of short knockdown cells (Figures S7A and S7B). Repeated, unsuccessful attempts prompted us to consider the possibility that it might not be the protein-coding function of the short isoform that is important. Interestingly, in addition to the 333 nt CDS, the endogenous ASCC3 short isoform transcript also contains a 2.8 kb 3′ untranslated region (3′-UTR), which is unique to this isoform (Figure 4A). To test the hypothesis that the function of the ASCC3 short mRNA isoform required the non-coding 3′ RNA sequence, we again expressed ASCC3 short isoform, this time including the 3′ sequence, which does not itself contain open reading frames (ORFs) of significant length. Importantly, the 13 kDa encoded protein was expressed to similar levels irrespective of inclusion of the 3′-UTR in the transcript (Figure S7B). Remarkably, however, in contrast to the CDS alone, the transcript containing the 3′-UTR suppressed the low transcription phenotype (Figures 7A and 7B).

These results suggest that the short ASCC3 isoform promotes transcription restart via a mechanism that is mediated by RNA, not protein. To further investigate this possibility, we assessed cells for expression of the protein encoded by the short ASCC3 isoform. Although the 13 kDa protein product of this isoform could be detected following ectopic expression using an antibody targeted toward its unique C terminus (Figure S7B), the protein could not be detected in untransfected cells. We therefore generated an antibody against an N-terminal epitope of ASCC3, which is shared between the long and short protein isoforms. Immunoprecipitation using this antibody pulled down the large (251 kDa) ASCC3 protein as well as the ectopically expressed 13 kDa isoform, but the endogenous short protein isoform could not be detected, neither by immunoblotting nor targeted mass spectroscopy (Figure S7C, and data not shown).

To more conclusively test whether ASCC3 short isoform was indeed functioning as a non-coding RNA, we now used the construct expressing the CDS with its 3′UTR, but this time inserting a premature stop mutation at the beginning of the CDS. As expected, this construct failed to produce protein (Figure S7B). Nevertheless, it rescued the low transcription phenotype in cells deficient for the short isoform (Figures 7A–7D), showing that the short ASCC3 isoform must function as a non-coding RNA.

RNA in situ hybridization experiments revealed that the short ASCC3 isoform transcript is overwhelmingly nuclear with some enrichment in discrete spots within the nucleus (Figure 7E). Localization was not significantly affected by UV irradiation, and the knockout cells lost the signal, confirming that the probes for in situ hybridization were specific (Figures S7D and S7E). In contrast, probes targeting the protein-coding long ASCC3 isoform produced a signal in both the nucleus and cytoplasm (Figure 7E). Biochemical cell fractionation producing cytoplasmic (S1), nucleoplasmic (S2), and chromatin-enriched (P2) fractions (Figure 7F) further revealed that the short ASCC3 isoform is primarily chromatin-associated (Figure 7G), similar to other long non-coding RNAs (lncRNAs), including MALAT-1 (Figure S7F).

Together, these data show that the short ASCC3 isoform functions as a non-coding RNA in the nucleus of human cells.

## Discussion

In this report, we provide evidence for a dramatic and global effect of UV irradiation on transcript elongation, which impacts RNA processing and provides significant potential for cellular regulation. UV exposure results in spatial restriction of transcription and slower elongation, with the result that only the promoter-proximal 20–25 kb are efficiently transcribed. Together, these events underlie a switch to expression of short mRNA isoforms and preferential use of alternative last exons in a number of genes, including *ASCC3*. Intriguingly, the switch between ASCC3 isoforms occurs on more than one level, in that the long mRNA isoform encodes a protein, functioning in the context of the ASCC complex and required for maintaining transcriptional suppression in the late stages of the DNA damage response, whereas the short isoform functions as a nuclear non-coding RNA that is required for transcription to recover. Intriguingly, the short and long isoforms constitute an autonomous regulatory module and functionally interrelate, so that the effect of deleting one can be at least partially compensated for by deleting the other (Figure 7H).

### Preferential Short ALE Isoform Expression in Response to Elongation Shutdown

The spatial restriction of transcription is surprising, but might allow some short genes to remain expressed after UV irradiation. Indeed, this phenomenon may finally explain the puzzling observation that human genes that remain expressed or are induced upon UV irradiation are invariably very short (McKay et al., 2004).

The significant spatial restriction of transcription activity and attenuation of elongation also explains the reduction in expression of long transcript isoforms, while the relative persistence of promoter-proximal RNA synthesis allows expression of short mRNA isoforms. Indeed, it seems obvious that region-restricted transcription, combined with slow transcript elongation, must underlie the increased expression of ALEs associated with these short RNA isoforms. Interestingly, data from others support the idea that recognition and inclusion of an ALE might slow transcription down even further (Kwak et al., 2013, Nojima et al., 2015) and thus promote the usage of otherwise dormant poly-A sites (Pinto et al., 2011). In this sense, ALE isoform expression might arguably also be classified as alternative termination/poly-adenylation (poly-A) events, due to the inherently different poly-A sites associated with these transcript isoforms.

### The Transcriptional Response to UV Irradiation Is Multi-layered and Complex

The analysis presented here uncovers an unexpectedly complex transcriptional response to UV exposure, as well as novel proteins and a non-coding RNA involved in regulating it. The transcription response can be sub-divided into several distinct phases. First, the immediate response to UV irradiation is a rapid and dramatic decrease in transcript elongation rates, within minutes of exposure. Second, this is followed by a decrease in transcriptional initiation within 2 hr of exposure. Together, these events constitute the molecular manifestation of the long established “global transcription shutdown” first observed decades ago (Mayne and Lehmann, 1982).

Third, a state of slow elongation is sustained for at least 12 hr following UV irradiation, despite the fact that lesion density is greatest immediately after UV irradiation and lesion removal in genes occurs at an exponential rate with a half-life of 8 hr after 15 J/m^2^ irradiation (Venema et al., 1990). This strongly suggests that the transcriptional response to UV irradiation is not caused solely by RNAPII stalling at DNA damage, but that UV irradiation also results in the activation of protein factors and pathways in *trans*. In support of this idea, our ongoing experiments with mutants from the screen for genes affecting transcription after DNA damage that also uncovered ASCC3 (Boeing et al., 2016), as well as recent data on *PRC1* and *UBR5* (Sanchez et al., 2016), strongly indicate that certain protein factors are indeed required for UV-induced transcription shutdown to take place. Without these factors, transcription continues even in the presence of DNA damage.

Fourth, as outlined in detail here, the widespread repression of transcription is maintained in the late phases of the UV-induced DNA damage response by a novel, separate mechanism, namely via ASCC complex-mediated transcriptional suppression. Interestingly, *ASCC3* is not required for the establishment of transcriptional repression, only for maintaining it. Remarkably, this intriguing suppression mechanism is negated by the action of the short ASCC3 RNA isoform, which ultimately allows transcription to recover.

### ALE Isoform Expression of ASCC3 Regulates the Transcription Response to UV Irradiation

Our data on *ASCC3* comprise evidence that the UV-induced shift to expression of short ALE transcript isoforms represents physiologically important regulation. Intriguingly, knockdown of the long ASCC3 isoform rescues the transcription defect in cells lacking the short isoform, highlighting that the long and short isoforms regulate one another to control transcription after UV irradiation. This indicates that the balance between long and short isoform expression, which is temporarily altered as a consequence of UV irradiation, is critical for regulating transcription shutdown and recovery.

Despite being annotated as protein-coding, the short ASCC3 transcript isoform is nuclear and may in fact not be translated to a significant extent. Indeed, its function in transcriptional restart after UV irradiation is dependent on the non-coding 3′ UTR and is retained after its coding ability is disrupted. The short ASCC3 RNA isoform likely functions as a non-coding RNA. Long non-coding RNAs (lncRNAs) are generally bioinformatically characterized by being relatively stable, RNAPII-generated RNAs lacking ORFs of 300 nts or more (Derrien et al., 2012). However, the distinction between mRNAs and lncRNAs is often somewhat blurred (Sampath and Ephrussi, 2016), and our data show that even though the short ASCC3 isoform does contain an ORF of 333 nts, it is functionally a lncRNA (of ∼3,500 bases). This points to a previously uninvestigated source of lncRNAs, namely alternative last exon (ALE)-derived, non-coding transcript isoforms produced from well-known protein-coding genes. To our knowledge, the only other example of a gene with alternative protein coding and functional lncRNA transcript isoforms is steroid receptor RNA activator 1 (*SRA*). Ironically, in contrast to ASCC3, *SR*A was long thought to encode a lncRNA, which regulates steroid hormone receptor driven transcription, but it may also produce ORF-containing alternative transcript variants that can be translated into protein. Unlike ASCC3, however, *SRA* produces alternative protein-coding splicing isoforms through mechanisms that introduce AUG codons not present in the lncRNA isoform (reviewed by Leygue, 2007).

The short ASCC3 RNA isoform appears to function, at least in part, by repressing the function of the ASCC complex, of which ASCC3 protein is a DEAD/DEAH box DNA helicase component (Jung et al., 2002, Dango et al., 2011). ASCC3/ASCC complex was identified through its role in transcriptional regulation (Jung et al., 2002, Li et al., 2013), but its biochemical mechanism of action remains unknown. We found that ASCC3 interacts with both RNAPII and CSB and it becomes highly ubiquitylated and phosphorylated upon UV irradiation (Boeing et al., 2016), suggesting a direct effect on transcription and regulation via post-translational modification. Understanding the biochemical function of ASCC complex is an important future goal, not least because it is a prerequisite for understanding the function of the ASCC3 lncRNA. Although we have so far failed to uncover convincing evidence for it, one possibility is that the chromatin-associated ASCC3 lncRNA regulates transcription through binding and regulating the ASCC complex. However, it might also function through recruitment of other factors. For example, lncRNAs such as HOTAIR and XIST both regulate transcription through recruitment of histone modification complexes and in the case of HOTAIR, even ubiquitin ligases (Bhan and Mandal, 2015, Rutenberg-Schoenberg et al., 2016). Two DNA damage-induced lncRNAs, lincRNA-p21 and PANDA, regulate p53-mediated gene expression by interacting with DNA/RNA binding proteins, resulting in gene-specific repression (Huarte et al., 2010, Hung et al., 2011). Post-transcriptional mechanisms for lncRNA function have also been described, including miRNA sequestering and regulating mRNA decay and translation (Abdelmohsen et al., 2013).

### Other UV-Induced ALE Genes

Intriguingly, our analysis uncovered a number of other genes with characteristics similar to those of *ASCC3.* For example, *INTS6* encodes an 887 amino acid (aa) protein, which is a subunit of the Integrator complex (Baillat et al., 2005). Upon UV irradiation, however, a much shorter RNA isoform is expressed, with the capacity to encode a 115 aa protein, which lacks the C-terminal region required for association with INTS3 and presumably the rest of the Integrator complex (Zhang et al., 2013). Likewise, *HERC4* encodes a putative ubiquitin ligase (1,057 aa), but also a short UV-induced isoform potentially encoding a 110 aa protein, which lacks the catalytic domain. Other interesting examples, such as *SUPT16H* (encoding the large subunit of the histone chaperone FACT) and *RAD51C* (involved in homologous DNA recombination) were also detected. Again, both encode very short, UV-induced isoforms, which might not result in functional proteins. Some of these short protein isoforms have been detected in a deep proteome sequencing project (Kim et al., 2014), but it is unclear whether they are functionally relevant, or whether, like for *ASCC3*, the short, stable, poly-adenylated transcript isoforms encoding them act in the form of lncRNAs. Addressing the precise function of these transcripts in the DNA damage response represents an important future goal.

## STAR★Methods

### Key Resources Table

**Table:** 

REAGENT or RESOURCE	SOURCE	IDENTIFIER
**Antibodies**		

RPB1	The Francis Crick Institute Core Facility	4H8
Tubulin	The Francis Crick Institute Core Facility	Tat-1
hnRNPA1	Abcam	Ab5832
Histone H3	Abcam	Ab1791
ASCC3 (long protein)	Dango et al., 2011	N/A
ASCC3 (short protein)	This Paper	N/A
ASCC3 (N-terminal)	This Paper	N/A

**Chemicals, Peptides, and Recombinant Proteins**		

5,6-Dichlorobenzimidazole 1-β-D-ribofuranoside (DRB)	Sigma-Aldrich	D1916
Anti-BrUTP, agarose coupled	Santa Cruz Biotech.	sc-32323 AC
5-Bromouridine 5′-triphosphate sodium salt (Br-UTP)	Sigma-Aldrich	B7166
iQ SYBR green supermix	Bio-Rad	1708880
Superase	ThermoFisher Scientific	AM2694
DNase, RNase Free	Promega	M6101
INTERFERin	Polyplus	409-10
5 Ethynyl-uridine	Jena Bioscience	CLK-N002-10
Alexa Flour 488 Azide	ThermoFisher Scientific	A10266
Dynabeads Protein A	ThermoFisher Scientific	10001D
ANTI-FLAG M2 Affinity Gel	Sigma-Aldrich	A2220
Methyl methanesulfonate (MMS)	Sigma-Aldrich	129925
*cis*-Diammineplatinum(II) dichloride (Cisplatin)	Sigma-Aldrich	P4394
Camptothecin	Sigma-Aldrich	CDS008734
ASCC3 peptide 1 for N-terminal antibody: MALPRLTGALRSFSNVTKQDNYNEC-CONH2	This paper	N/A
ASCC3 peptide 2 for N-terminal antibody: KRSKLHEQVLDLGC- CONH2	This paper	N/A
ASCC3 peptide for Short isoform antibody CPFQKRRLDGKEEDEKMSRASDRFRGLR-COOH	This paper	N/A

**Critical Commercial Assays**		

Nuclei Isolation Kit: Nuclei EZ Prep	Sigma-Aldrich	NUC101-1KT
RNeasy Mini Kit	QIAGEN	74104
miRNeasy Mini Kit	QIAGEN	217004
TruSeq RNA Sample Preparation kit	Illumina	RS-930-2001
TruSeq Stranded Total RNA LT Sample Prep kit	Illumina	RS-122-2201/2
Taqman Reverse Transcriptase Reagents	Applied Biosystems	N8080234
RNAscope 2.5 HD Reagent Kit-RED	Advanced Cell Diagnotics	322350
RNAscope Probe- Hs-ASCC3-tv1	Advanced Cell Diagnotics	468231
RNAscope Probe- Hs-ASCC3-tv2	Advanced Cell Diagnotics	443331

**Deposited Data**		

DRB/GRO-Seq -/+ UV	This study	GEO: GSE91010
UV 24 hr GRO-Seq	This study	GEO: GSE91011
RNASeq -/+ UV 8 and 24 hr	This study	GEO: GSE92239
Illumina bead array -/+ UV ASCC3 short isoform knockout cells	This study	GEO: GSE92325
Illumina bead array -/+ UV ASCC3 long isoform shRNA knockdown cells	This study	GEO: GSE92327

**Experimental Models: Cell Lines**		

Human: MRC5VA cell line	The Francis Crick Institute Cell Services	N/A

**Experimental Models: Organisms/Strains**		

Human: MRC5VA shASCC3 (long)	The Francis Crick Institute Cell Services	N/A
Human: MRC5VA ASCC3 short KO clones 1 and 2	The Francis Crick Institute Cell Services	N/A

**Recombinant DNA**		

pSpCas9(BB)-2A-GFP (PX458)	Addgene	48138
pTre3G (empty plasmid)	Clontech	631173
pTre3G Flag ASCC3 short coding sequence (CDS) cDNA	This paper	N/A
pTre3G Flag ASCC3 short CDS with 3′UTR cDNA	This paper	N/A
pTre3G Flag ASCC3 short Mutated CDS with 3′UTR cDNA	This paper	N/A

**Sequence-Based Reagents**		

See Table S5		

**Software and Algorithms**		

BWA	Li and Durbin, 2009	http://maq.sourceforge.net/
SAMtools	Li et al., 2009	http://samtools.sourceforge.net/
TopHat2	Kim et al., 2013	http://ccb.jhu.edu/software/tophat/index.shtml
MISO	Katz et al., 2010	http://genes.mit.edu/burgelab/miso/
DEXSeq	Anders et al., 2012	http://bioconductor.org/packages/release/bioc/html/DEXSeq.html
GenomicRanges	Lawrence et al., 2013	https://bioconductor.org/packages/release/bioc/html/GenomicRanges.html
Limma	Ritchie et al., 2015	https://bioconductor.org/packages/release/bioc/html/limma.html
DAVID Bioinformatics resource	Huang et al., 2009	https://david.ncifcrf.gov/
HCS Studio 2.0 Cell Analysis Software	ThermoFisher Scientific	https://www.thermofisher.com/order/catalog/product/SX000041A

### Contact for Reagent and Resource Sharing

For reagent requests please contact Jesper Svejstrup (jesper.svejstrup@crick.ac.uk).

### Experimental Model and Subject Details

Human MRC5VA cells were grown at 37°C, 5% CO_2_ in DMEM supplemented with 10% fetal bovine serum and 5% penicillin/streptomycin. Cell lines were routinely screened for mycoplasma contamination and human species authenticated by STR profiling and PCR based analysis, performed by the Francis Crick Institute Cell Services.

### Method Details

#### Cell line manipulation and generation

Cells were transfected with siRNAs using Interferin transfection reagent followed by a minimum of 48 hr incubation prior to UV irradiation. Lipofectamine 2000 was used for plasmid DNA transfection. For generation of stable ASCC3 knockdown cells, MRC5VA cells were infected with lentiviral particles carrying ASCC3 long isoform-targeting shRNA or non-targeting shRNA followed by selection with puromycin (1 μg/mL). For generation of ASCC3 short isoform knockout cells, cells were transfected with a Cas9- and guide RNA-containing vector (PX458) that targeted the introns up- and downstream of the terminal exon of the short isoform of ASCC3. Individual GFP-positive cells were selected by fluorescence-assisted cell sorting and clones were screened for deletion of the desired fragment by genomic PCR and RT-qPCR.

UVC-irradiation was performed using either a Stratalinker 2400 (Stratagene), or a purpose-built UVC box that was used to irradiate 384-well plates. Unless otherwise stated, 15 J/m^2^ was used.

#### GRO-Seq

UV/DRB/GRO-Seq (Figures 1A–1D, S1A, and S1B) was done essentially as described in Saponaro et al. (2014): approximately 6 X 10^6^ MRC5VA cells were cultured in DMEM media containing 10% FBS, 5% penicillin/streptinomycin and 100 μM 5,6-Dichlorobenzimidazole 1-β-D-ribofuranoside (DRB) for 3.5 hr. DRB-containing media was removed and cells were either left untreated or exposed to 15 J/m^2^ UVC irradiation. Cells were then washed with PBS and placed in fresh media without DRB. Cells were then incubated for 10, 25 or 40 min. Transcription-competent nuclei were prepared using the Nuclei Isolation Kit by scraping cells in 10 mL of cold lysis buffer followed by a spin at 500 x g for 5 min at 4°C then resuspended in 400 μL cold storage buffer supplemented with protease inhibitors and Superase. Nuclear Run-On reactions were carried out by addition of 400 μL Run-On Buffer (10 mM Tris-Cl pH 8.0, 5 mM MgCl_2_, 1 mM DTT, 300 mM KCL, 20 units of SUPERase, 1% Sarkosyl, 500 μM ATP, GTP, CTP and Br-UTP) and incubation at 30°C for 5 min. Run-On reactions were stopped by addition of 10 X DNaseI buffer (93 μL) and RNase Free DNase (40 μL) and incubation for 1.5 hr 30°C shaking. Br-UTP run-on labeled RNA was isolated using anti-Br-UTP coupled agarose beads at room temperature for 1 hr. Beads were washed once with low salt buffer (0.2X SSPE, 1mM EDTA, 0.05% Tween), twice with high salt buffer (0.5X SSPE, 1 mM EDTA, 0.05% Tween, 150 mM NaCl) and twice with TE pH 8.0 + 0.05% Tween. RNA was eluted from beads (20 mM DTT, 300 mM NaCl, 5 mM Tris pH7.5, 1 mM EDTA, 0.1 mg/mL glycogen and 0.1% SDS) at room temperature. Eluates were acid phenol-chloroform extracted and precipitated.

The purified RNA was used for the preparation of strand-specific RNA libraries using the TruSeq Stranded Total RNA LT Sample Prep kit, and sequenced on an Illumina HiSeq 2000 sequence analyzer as single-ended 51 bp reads.

For the 0-24 hr GRO-Seq experiment (Figures 1E, 1F, and S1C–S1F), approximately 6 X 10^6^ of MRC5VA cells were either left untreated or UVC-irradiated with 15 J/m2 and allowed to recover for 2, 5, 8, 10, 12 or 24 hr. Nuclei were isolated and run-on and RNA isolation was performed as above.

The purified RNA was used for the preparation of strand-specific RNA libraries using the TruSeq Stranded Total RNA LT Sample Prep kit, and sequenced on an Illumina HiSeq 2000 sequence analyzer as 101 bp reads.

#### RNA-Seq

MRC5VA cells were either left untreated or treated with 15J/m^2^ of UVC irradiation followed by recovery for 8 or 24 hr. RNA was extracted using RNeasy Kit and analyzed on a 2100 Bioanalyzer (Agilent Technologies). All samples had an RIN value of greater than 8. The purified RNA was used for the preparation of poly A selected mRNA libraries using the TruSeq RNA sample preparation kit and sequenced on an Illumina GA IIX sequence analyzer as paired-end 72 bp reads.

#### Reverse Transcriptase Quantitative PCR

RNeasy Mini Kit purified RNA was used to generate random hexamer primed cDNA libraries using Taqman Reverse Transcriptase Reagents. Quantitative PCR were performed using iQ SYBR green Mastermix, 0.3 μM primer concentration and equal 1 μL of cDNA library per reaction. Reference gene normalized RNA expression was compared between variable (ex. UV-treated) and control samples using the Livak equation (Livak and Schmittgen, 2001). For example, to measure the expression of the short isoform of ASCC3 under UV-treated compared to untreated conditions, the following equation was used (2^∧^-((CT_ASCC3short_-CT_GAPDH_)_UV_-(CT_ASCC3short_-CT_GAPDH_)_UN_)).

For experiments where RNA levels were compared between subcellular fractions (Figures 7G and S7F), ASCC3 short isoform and Malat-1 CT values were not normalized to a reference gene. Here, relative RNA levels in the nucleoplasmic enriched S2 fraction compared to cytoplasmic S1 fraction was calculated using the following Equation (2^∧^-(CT_S2fraction_-CT_S1fraction_). RNA levels in the chromatin fraction were similarly determined using (2^∧^-(CT_P3fraction_-CT_S1fraction_). Column graphs represent the average relative RNA expression across at least 3 biological replicate experiments. Primer sequences are found in Table S5.

#### 5′ Ethynyl Uridine transcription assay

SiRNA was diluted in HBSS (500 nM) and 5 μL of diluted siRNA was deposited in 384 well plates. Interferin transfection reagent was diluted 1/100 in OPTI-MEM and 5 μL of transfection reagent was deposited on top of diluted siRNA. SiRNA/Interferin mix was incubated at room temperature for 15 min. MRC5VA cells were diluted in DMEM containing 10% FBS and 5% penicillin/streptinomycin to a density of 2.25 X 10^4^ cells/mL and 40 μL was deposited on top of the transfection mix (900 cells/well). Cells were incubated for 48 hr. Media was aspirated from the 384-well plates and cells were either exposed to 15 J/m^2^ using a custom built UVC box or left untreated. Media was replaced and cells were incubated for the indicated amount of time. Media was replaced with fresh media containing 0.75 mM 5 Ethynyl uridine (EU) and cells were incubated for 2 hr. EU-containing media was removed and cells were fixed in PBS buffered formaldehyde (3.7%) for 45 min at room temperature, washed once with PBS using a plate washer followed by permeabilization with 0.5% TX-100 diluted in PBS for 30 min. Plates were washed once with PBS then Alexa Fluor 488 Azide fluorophores were covalently attached to EU-containing RNA by click reaction (100 mM Tris pH 8.5, 4 mM Cu_2_SO_4_, 10 μM Alexa azide 488, 100mM Ascorbic Acid) for 1 hr at room temperature. Plates were washed 3 times in 100 mM Tris, pH 7.5 and stained with DAPI (4′,6-diamidino-2-phenylindole dihydrochloride) at a final concentration of 1 μg/ml. Plates were washed once with PBS. Automated image acquisition of 6 fields per well was performed (Cellomics Array Scan VTI, ThermoFisher Scientific) using a 10 × objective.

#### Clonogenic Survival Assay

1.5 X 10^5^ MRC5VA cells were cultured in 6-well dishes and incubated overnight. SiRNA targeting ASCC3 or non-targeting control was diluted in 150 μL HBSS (500 nM) and mixed with Interferin diluted in 150 μL OPTI-MEM (5 μL interferin per well). The siRNA/Interferin mix was incubated at room temperature for 15 min then added to wells containing 2.7 mL of fresh media (final siRNA concentration 50 nM). Plates were incubated for 30 hr then cells were treated with Trypsin/EDTA, counted and seeded in 6-cm dishes at a low density for colony formation. Cells were incubated overnight followed by UVC irradiation with 2, 5, 10 and 15 J/m^2^ (48 hr after siRNA transfection). Colonies were allowed to form over a 10-14 day period and were stained with crystal violet and counted.

#### Gene expression array

RNA was purified using RNeasy Mini Kit from ASCC3 short isoform knockout (clone 2) cells and MRC5VA parental control cells, ASCC3 long isoform knockdown cells and scrambled shRNA control cells either in untreated conditions or 20 hr following UV irradiation. Each condition was represented by 3 biological replicates. RNA samples were processed by services provided at the Oxford Genomic Centre using the Illumina TotalPrep-96 RNA Amplification Kit followed by the Illumina Whole-Genome Gene Expression Direct Hybridization Assay. The labeled cRNA was then hybridized to Human HT-12_V4_BeadChip for gene expression.

#### Subcellular fractionation

Protocol was adapted from Mayer et al. (2015). MRC5VA cells were left untreated or UV-irradiated followed by 4 hr or 20 hr recovery. ∼4 x10^6^ cells/ condition were harvested by trypsin/EDTA, washed with PBS, resuspended in Cytoplasmic lysis buffer (0.15% NP-40, 10 mM Tris-HCl (pH 7.0), 150 mM NaCl, 50 U SUPERase, 1 X protease inhibitor mix) and incubated on ice 5 min. Lysates were layered on top of Sucrose buffer (10 mM Tris-HCl (pH 7.0), 150 mM NaCl, 25% sucrose, 50 U SUPERase, 1 X protease inhibitor mix) and centrifuged 16000 g 10 min 4°C. The supernatent was collected for the cytoplasmic fraction and pelleted nuclei were washed in Nuclei wash buffer (0.1% Triton X-100, 1 mM EDTA, in 1x PBS, 50 U SUPERase and 1x protease inhibitor mix) spun at 1150 g for 1 min then resuspended in Glycerol buffer (20 mM Tris-HCl (pH 8.0), 75 mM NaCl, 0.5 mM EDTA, 50% glycerol, 0.85 mM DTT, 50 U SUPERase and 1x protease inhibitor mix). Equal volume of Nuclei lysis buffer (1% NP-40, 20 mM HEPES pH 7.5, 300 mM NaCl, 1M Urea, 0.2 mM EDTA, 1 mM DTT, 50 U SUPERase and 1x protease inhibitor mix) was added followed by vortex and 2 min incubation on ice. Chromatin was pelleted by centrifugation at 18500 g for 2 min 4°C and supernatent collected as the Nuclear fraction. Chromatin pellets were resuspended in DNaseI buffer (40 mM Tris pH8.0, 10 mM MgSO_4_, 1 mM CaCl_2_, 50 U Superase In, 10 U RNase Free DNase) followed by 30 min incubation at 37°C. Digestions were centrifuged 18500 g 10 min and chromatin supernatents were collected. An aliquot of Cytoplasmic, Nuclear and Chromatin fractions were reserved for immunoblot analysis and RNA was purified from the remaining sample using miRNeasy Mini Kit. CDNA libraries generated from purified RNA were subjected to qPCR using primers specific for the short isoform of ASCC3 and Malat-1.

#### Ectopic expression of ASCC3 short isoform constructs

MRC5VA cells stably expressing the CMV-Tet3G transactivator protein were transfected using lipofectamine 2000 with pTre3G vector constructs containing siRNA resistant N-terminally Flag-tagged ASCC3 short isoform coding sequence (CDS), CDS with 3′ UTR or CDS containing an in-frame stop mutation with 3′UTR. In experiments where endogenous ASCC3 short isoform was knocked down, 24-32 hr after transfection with ASCC3 transgene expression constructs, cells were subsequently transfected with ASCC3 short isoform targeting or NT siRNA followed by addition of doxycycline (100 ng/mL) for 24 hr to support transgene expression. For experiments involving knockout cell lines, transgene transfected cells were cultured in the presence of doxycycline (100 ng/mL) for at least 24 hr. Cells were subsequently exposed to UV irradiation or left untreated and incubated for 20 hr followed by EU incorporation assay.

#### Immunoprecipitation/Immunoblotting

Expression of the corresponding short isoform protein in Figures S7B and S7C was determined by immunoprecipitation after transient transfection with the indicated constructs in the presence of doxycycline (100 ng/mL) followed by immunoblotting. Whole cell lysates were prepared 24 hr after transfection by lysis in cell lysis buffer (50 mM HEPES pH 7.9, 150 mM NaCl, 3 mM MgCl2, 10 mM EDTA, 10% glycerol, 1% NP-40) followed by immunoprecipitation using M2 Flag agarose or ASCC3 N-terminal-targeting antibody for 4 hr. ASCC3 N-terminal antibody was immobilized on protein A Dynabeads. Bead bound immunoprecipitations were washed 3 times in lysis buffer and eluted by boiling in 2 X laemmli sample buffer.

For immunoblotting, protein samples were separated by SDS-PAGE on 4%–12% polyacrylamide gels and transferred onto nitrocellulose membrane. Membranes were probed using the indicated antibodies.

#### RNA in situ hybridization

RNASCOPE 2.5 HD Assay Red Kit and probes targeting the long and short isoform of ASCC3 were designed and supplied by Advanced Cell Diagnostics. Cells were grown on slides and either left untreated or UV-irradiated with 15 J/m2 followed by 20 hr incubation. Cells were washed in PBS, fixed in 10% Neutral Buffered Formalin for 30 min. Fixed cells were then washed with PBS followed by dehydration in ethanol according to manufacturers protocol. Following dehydration, slides were stored in 100% ethanol at −20 C. Probe hybridization was performed strictly according to manufacturers protocol and counterstained with DAPI. Images were acquired using a Leica TCS SP5 confocal microscope.

### Quantification and Statistical Analysis

#### GRO-Seq analysis

51 bp single-end reads (DRB/GRO-Seq) and 101 bp single-end reads (24 hr GRO-Seq) were aligned to the hg19 genome assembly using BWA v0.5.9 and v0.7.10 (Li and Durbin, 2009) respectively with default settings. BAM files were sorted and indexed using SAMtools (Li et al., 2009). Further analysis was conducted using Bioconductor and its GenomicRanges package (Lawrence et al., 2013). Reads were extended to 250 bp and each sample was normalized to a read depth of 20 million.

A subset of the protein coding human Ensembl transcriptome was created by filtering for transcripts ≥ 30 kb. The largest transcript per gene was selected, resulting in a list of 8,148 transcribed genes. Base pair level coverage of the region 2 kb upstream, to 120 kb downstream, of each transcript’s TSS was calculated for each sample. Average transcript profiles were generated by taking a trimmed mean (0.05) of read depth over each base pair.

#### Gene specific wave-fronts and elongation rate for DRB/GRO-Seq

Islands of normalized read depth ≥ 3 bp were identified for each gene from the TSS to 120 kb downstream. These islands were assumed to be evidence of elongation. When read-depth first dropped below 3 for 5,000 consecutive base pairs, elongation was assumed to have halted, and a wave front was called at the transition point. The 5 kb distance filter was necessary to filter out background noise and downstream transcripts. Short exonic regions were also excluded in the wave front calling analyses. The subsequent list of wave fronts was filtered to remove genes that did not have an increase in wave front progression over time and that had a wave front called downstream of the transcription termination site. The resulting filtered list was manually curated further to remove genes with interfering antisense and convergent transcription resulting in a list of 333 genes with wave fronts calculated for all untreated and UV-treated time points. These were plotted and the median wave front was determined for each sample. To calculate elongation rates, the difference in wave front position between the 10, 25 and 40 min time points was calculated for each gene then divided by the 15 min time interval.

#### Gene specific wave-fronts for GRO-Seq

Single gene GRO-Seq wave fronts were determined for UV-treated 2-24 hr GRO-Seq experiment (Figures S1D and S1E). Wave fronts were computationally determined as above. The resulting list of wave fronts were filtered to include expressed genes (RPKM > = 0.3 0 to +250 bp relative to TSS) that displayed sustained GRO-Seq signal between +30 kb (relative to the TSS) and 1 kb downstream of the TTS. We focused on genes that displayed a reduction in wave front position in response to UV. This resulted in a list of 141 genes with wave fronts calculated for all samples in the UV time course. These were plotted and the median wave front was determined for each sample. The elongation rate 2-12 hr post-UV was determined by plotting the median wave front over time and including a line of best fit. The slope of the line indicates the elongation rate.

#### Mathematically determined wave-fronts for GRO-Seq

The average gene profile for each of the UV-treated samples was normalized by subtracting from the untreated average gene profile. The resulting normalized UV-treated profile was smoothed using a loess line. The position along the x axis at which the loess line crosses y = 0 was used as an estimate of wave-front position. This estimate was further refined by taking the first instance after the initial wave-front estimate where the derivative of the fitted line was half that at the position of the initial estimate. The elongation rate 2-12 hr post-UV was determined by plotting the median wave front over time and including a line of best fit (Figure S1F). The slope of the line indicates the transcription elongation rate.

#### Single-gene GRO-Seq profiling

A bp resolution profile of log2 (1+ normalized read depth) was created for a single gene’s genomic range ± 2kb. The range was divided into consecutive 500 bp bins and a mean coverage depth across each bin was calculated. A smoothing spline was then applied.

#### Terminal exon synthesis analysis

The RPKM of GRO-Seq signal corresponding to the TSS distal and proximal terminal exons of long and short alternative transcript isoforms was calculated respectively. The UV-treated RPKM for terminal exons corresponding to short and long transcript isoforms was normalized to untreated conditions and log2 transformed. The ratio of normalized TSS proximal terminal exon log2(RPKM) to TSS distal log2(RPKM) was calculated for UV regulated ALE events as well as and background events that were not regulated by UV irradiation and plotted to determine the ratio of short to long isoform expression (Figures 2G and S2D–S2F). Statistical analysis in S5D is Mann Whitney test, ^∗∗^ p < 0.01, ^∗∗∗∗^ p < 0.0001.

#### RNA-Seq alternative isoform analysis

RNASeq reads were aligned to UCSC genome hg19 using Tophat2 (Kim et al., 2013). Transcript information was obtained from the UCSC known Gene table. Ambiguously mapped reads were removed from the alignments prior to subsequent analysis (mapped read-pair range 17111950 - 27119645).

We identified differential spicing events using MISO (Katz et al., 2010) along with MISO’s published splicing event annotation. MISO was run against pairwise combinations of UV-treated and untreated samples for two biological replicates. We filtered results using MISO’s post filtering script to generate final events (The number of both inclusion and exclusion reads > 10, delta-psi > 0 and bayes-factor > 10). Statistically significant events called for the same UV time point in both biological replicates were filtered further for splicing effect size. To determine an effect size for the different splicing events we quantified each event at the exon level (Splicing Index (SI)). For events affecting a single exon or intron (Skipped Exon (SE), Retained Intron (RI)), we quantified the spicing event as a ratio of exon/intron mapped reads over gene mapped reads (For example, for the skipped exon category log2((Exon_UV_/Gene_UV_)/(Exon_UN_/Gene_UN_)). In the cases of alternative exon usage where splicing is indicated by a shift from one exon to another (Alternative first/last exon usage (AFE, ALE)), we quantified the events as a ratio of reads mapping to one over the other (log2((Exon2_UV_/Exon2_UN_)/(Exon1_UV_/Exon1_UN_)). For alternative splice site events (A5SS, A3SS) where a proportion of the alternative exon is shared between the two events we used only reads unique to each event. These counts were treated as in the AFE/ALE cases above. Only splicing events that had a log2(SI) of ≥ 0.25 or ≤ −0.25 in both biological replicates were included in our final list of UV-induced splicing events (Table S1). Table S2 shows additional MISO output parameters PSI, deltaPSI and Bayes factor for UV-regulated ALE events.

Non-UV regulated ALE background events (Figure S2) are defined as all potential ALE events in the MISO database of splicing event annotation that were not significantly affected by UV treatment (n = 7736).

DEXSeq (Anders et al., 2012) was used to produce the exon level expression plots. Plots were manually edited to restrict the analysis to exons present in the isoforms of interest.

#### Gene ontology analysis

Gene ontology analysis was performed by uploading gene symbols for UV-regulated ALE events to DAVID Bioinformatics Resources 6.7 (Huang et al., 2009).

#### Gene expression array

Background subtracted probe signals were averaged across the 3 biological replicates and processed using Limma Bioconductor package. Genes that were downregulated with a fold change ≤ −1.5 fold in UV-treated ASCC3 short isoform knockout cells compared to UV-treated parental cells were subjected to hierarchical clustering and log2 fold change values for both short isoform knockout and shASCC3 long isoform cells were displayed in a heatmap. Results for all genes are shown in Table S4.

#### EU Assay

Image analysis was performed using HCS Studio 2.0. Cell nuclei were masked using the DAPI staining. The average intensity of Alexa Fluor 488-conjugated EU-labeled RNA was measured for each nucleus in at least 3 separate wells and plotted in a histogram. The threshold identifying low transcription was set for each experiment manually based on the histogram profiles and the percentage of cells below the threshold was calculated. Histograms represent one biological replicate consisting of 3 technical replicates (2500 - 20000 cells per condition) and column graphs with statistics represent the average of ≥ 3 biological replicates.

### Data and Software Availability

#### Software

##### Data Resources

The accession number for the DRB/GRO-Seq, 24 hr GRO-Seq, RNA Seq and illumina Bead Array data reported in this paper is GEO: GSE91012 https://www.ncbi.nlm.nih.gov/geo/query/acc.cgi?acc=GSE91012.

## Author Contributions

Conceptualization, L.W. and J.Q.S.; Methodology, L.W., M.S., S.B., and J.Q.S.; Formal Analysis, S.B., P.E., R.M., G.K., and A.L.; Investigation, L.W., M.S., T.K., J.W., and B.S.-D.; Writing – Original Draft, J.Q.S. and L.W.; Writing – Review & Editing, J.Q.S. and L.W.; Funding Acquisition, J.Q.S.; Supervision, J.Q.S., M.H., and A.S.

## Supplemental Information

Table S1. List of UV-Induced Alternative Splicing Events, Related to Figure 2 Table shows the time at which the event was detected, gene symbol, splicing event identification and the average splicing index. The average splicing index (SI) for the two biological replicates (as described in the STAR Methods) is shown as a measure of effect size.

Table S2. List of Alternative Last Exon Splicing Events, Related to Figure 2 This table shows the all UV-regulated Alternative Last Exon (ALE) splicing events. The time after UV, event name, gene name, TSS coordinate, and terminal exon coordinates for the ALE isoforms are shown. The “Exon 2 downstream of Exon 1” column designates if exon 2 corresponds to the long isoform (1) or short isoform (-1). The short isoform mean percent spliced in (PSI) values, delta PSI, and Bayes factor for each replicate and the average across both biological replicates is shown.

Table S3. UV-Induced ALE Short Events that Were Also Identified in the Transcription-Related DNA Damage Response Candidate Gene Database BioLogic, Related to Figure 2 Table shows the RNAi screen high and low transcription z scores, RNAPII-IP, CSB-IP, CSB-IP + proteasome inhibitor MG132, chromatin proteome and chromatin proteome + MG132 SILAC z scores for ALE Short genes identified in the BioLogic database. Positive SILAC z scores indicate increased interaction with RNAPII/CSB or increased association with chromatin after UV treatment, whereas negative SILAC z scores indicate a loss of interaction or chromatin association. The overall BioLogic Score is also shown. See Boeing et al. (2015) for full experimental details. N/A indicates the protein was not identified in the mass spec experiment.

Table S4. Gene Expression Array Results, Related to Figure 6 Gene symbols, background subtracted probe intensities, log fold changes and corresponding adjusted P values for the indicated comparisons are shown. Signal for untreated and UV-treated (15 J/m2, 20 hour recovery) knockout (KO) and matched Parental cells, shRNA targeting ASCC3 long isoform cells (shASCC3) and matched shRNA scrambled control cells are shown.

Table S5. siRNA, shRNA, and Primer Sequences, Related to the STAR Methods Target Sequences corresponding to the indicated siRNA and shRNA and guide RNA reagents and primer sequences are shown.
